# Structural basis of different neutralization capabilities of monoclonal antibodies against H7N9 virus

**DOI:** 10.1128/jvi.01400-24

**Published:** 2024-12-20

**Authors:** Bingbing Zhao, Zhenzhao Sun, Shida Wang, Zhibin Shi, Yongping Jiang, Xiurong Wang, Guohua Deng, Peirong Jiao, Hualan Chen, Jingfei Wang

**Affiliations:** 1 State Key Laboratory for Animal Disease Control and Prevention & National Data Center for Animal Infectious Diseases, Harbin Veterinary Research Institute, Chinese Academy of Agricultural Sciences 687216 , Harbin, People's Republic of China; 2 College of Veterinary Medicine, South China Agricultural University 554665 , Guangzhou, People's Republic of China; University of North Carolina at Chapel Hill, Chapel Hill, North Carolina, USA

**Keywords:** H7N9, HA, neutralizing antibody, cryo-EM structure, epitope, neutralizing mechanism

## Abstract

Neutralizing antibodies (nAbs) are important for the treatment of emerging viral diseases and for effective vaccine development. In this study, we generated and evaluated three nAbs (1H9, 2D7, and C4H4) against H7N9 influenza viruses and found that they differ in their ability to inhibit viral attachment, membrane fusion, and egress. We resolved the cryo-electron microscopy (cryo-EM) structures of H7N9 hemagglutinin (HA) alone and in complex with the nAb antigen-binding fragments (Fabs) and identified the HA head-located epitope for each nAb, thereby revealing the molecular basis and key residues that determine the differences in these nAbs in neutralizing H7N9 viruses. Moreover, we found that the humanized nAb CC4H4 provided complete protection in mice against death caused by a lethal H7N9 virus infection, even when nAb was given 3 days after the mice were infected. These findings provide new insights into the neutralizing mechanism and structural basis for the rational design of H7N9 virus vaccines and therapeutics.

**IMPORTANCE:**

H7N9 viruses have caused severe infections in both birds and humans since their emergence in early 2013 in China. Their persistent presence and variation in avian populations pose a significant threat to both poultry and humans. There are no treatments for human infections. In this study, we thoroughly investigated the neutralization mechanisms, structural basis, and therapeutic effects of three nAbs (1H9, 2D7, and C4H4) against H7N9 viruses. We revealed the molecular determinants underlying the varied performances of the three nAbs in neutralizing H7N9 viruses and protecting H7N9-infected mice. These insights provide a solid foundation for the rational design of vaccines and therapeutics against H7N9 viruses.

## INTRODUCTION

The continued evolution of influenza viruses leads to the emergence of antigenic variants that can escape from the host’s immunity either obtained through vaccination or a previous infection (1). Timely updates to vaccine strains are essential for controlling outbreaks caused by such variants; however, the delayed identification of the variant and the time-consuming vaccine development process present challenges to this effort (2). Therefore, the development of universal vaccines and broad-spectrum therapeutics is the key to the fight against antigenic variants. The hemagglutinin (HA) protein of the influenza virus is responsible for receptor engagement and is, therefore, one of the major targets for studying the virus-neutralizing mechanism and screening neutralizing antibodies (nAbs) against the virus (3–5). HA is initially synthesized as a precursor protein, HA0, and is conveyed along the constitutive secretory route to the plasma membrane (6, 7). The cleavage of HA by host cell proteases occurring in the endosomes is essential for the infectivity of the influenza virus. The cleavage site of low pathogenicity influenza viruses is constituted by an arginine residue (Arg343 in H1 numbering) and can be recognized by Plasmin, factor Xa, tryptase Clara, HAT, TMRPSS2, etc (8, 9). In contrast, the cleavage site of highly pathogenic influenza viruses consists of multiple basic amino acids and can be recognized by the furin protease that is ubiquitously expressed in the host (8, 9). After binding to the host cell receptor and being internalized into the endosome, the HA protein of the influenza virus is cleaved into HA1 and HA2, which are covalently linked by a disulfide bond (10). Subsequently, it undergoes a low-pH-induced, irreversible conformational change that is required for viral entry through membrane fusion (11). The neutralization mechanism of the HA-targeted nAbs involves inhibiting key steps in the virus life cycle, such as attachment, membrane fusion, and budding (12–15). Efforts to screen for nAbs against antigenic variants have revealed that nAbs that engage an epitope near the cleavage site of the HA can neutralize influenza viruses across subtypes (16–21). However, neutralization-escaping strains of those nAbs have also been reported, and the underlying mechanisms are not yet fully understood. More importantly, the antigenicity of newly emerged influenza viruses, such as the avian influenza A (H7N9) virus, as well as the neutralization mechanism of nAbs against these viruses, remains unclear, thereby hindering the development of effective vaccines and therapeutics.

Human infections with H7N9 viruses were first confirmed in February 2013 in several provinces of China (22). Since then, five waves of human infections have occurred, resulting in a total of 1,568 cases and 615 deaths (23). Initially, H7N9 viruses were low pathogenic in poultry. However, the continuous evolution of the viruses has led to the occurrence of more than 24 genotypes between 2013 and 2018 (24, 25). Notably, highly pathogenic H7N9 viruses, which are characterized by the polybasic amino acid insertions at the cleavage site of HA, emerged in early 2017, causing disease outbreaks in many chicken farms and an increased fatality rate among human cases (26). To eliminate this increasing threat, a vaccine has been approved and broadly used in chicken populations in China since September 2017, which stopped further human infections (25, 27). However, H7N9 viruses have not been eradicated and antigenic variants have been isolated continuously from poultry populations (28, 29). These antigenic variants escape from neutralization by the previously screened nAbs, presenting a persistent threat to avian and human populations.

In this study, we screened three murine nAbs (1H9, 2D7, and C4H4) against H7N9 viruses and explored their neutralization mechanisms. We resolved the cryo-electron microscopy (cryo-EM) structures of the HA trimer in complex with the antigen-binding fragments (Fabs) of the three nAbs and determined the molecular basis of their different neutralizing abilities. We evaluated the therapeutic effects of two humanized nAbs, C2D7 and CC4H4, for the treatment of H7N9 infection in mice. Our findings provide insights into the neutralization mechanisms of these nAbs against H7N9 viruses and may contribute to the rational design of vaccines and therapeutics targeting H7N9.

## RESULTS

### Binding and neutralization of H7N9 viruses by the nAbs

By immunizing mice with three H7N9 strains sequentially, we obtained three nAbs, 1H9, 2D7, and C4H4, that neutralize these three H7N9 strains with high efficiency. We then determined their amino acid sequences (Fig. S1A). To explore their neutralizing capacity against other H7N9 viruses, we performed an entropy analysis on 1,305 H7N9 HA amino acid sequences and selected five representative strains (A/pigeon/Shanghai/S1069/2013 [S1069], A/duck/Guangxi/SDY129/2014 [SDY129], A/chicken/Guangdong/SD008/2017 [SD008], A/chicken/Yunnan/SD193/2017 [SD193], and A/duck/Fujian/SD001/2018 [SD001]) to conduct subsequent tests. The phylogeny of these five viruses is shown in Fig. S1B.

**Fig 1:**
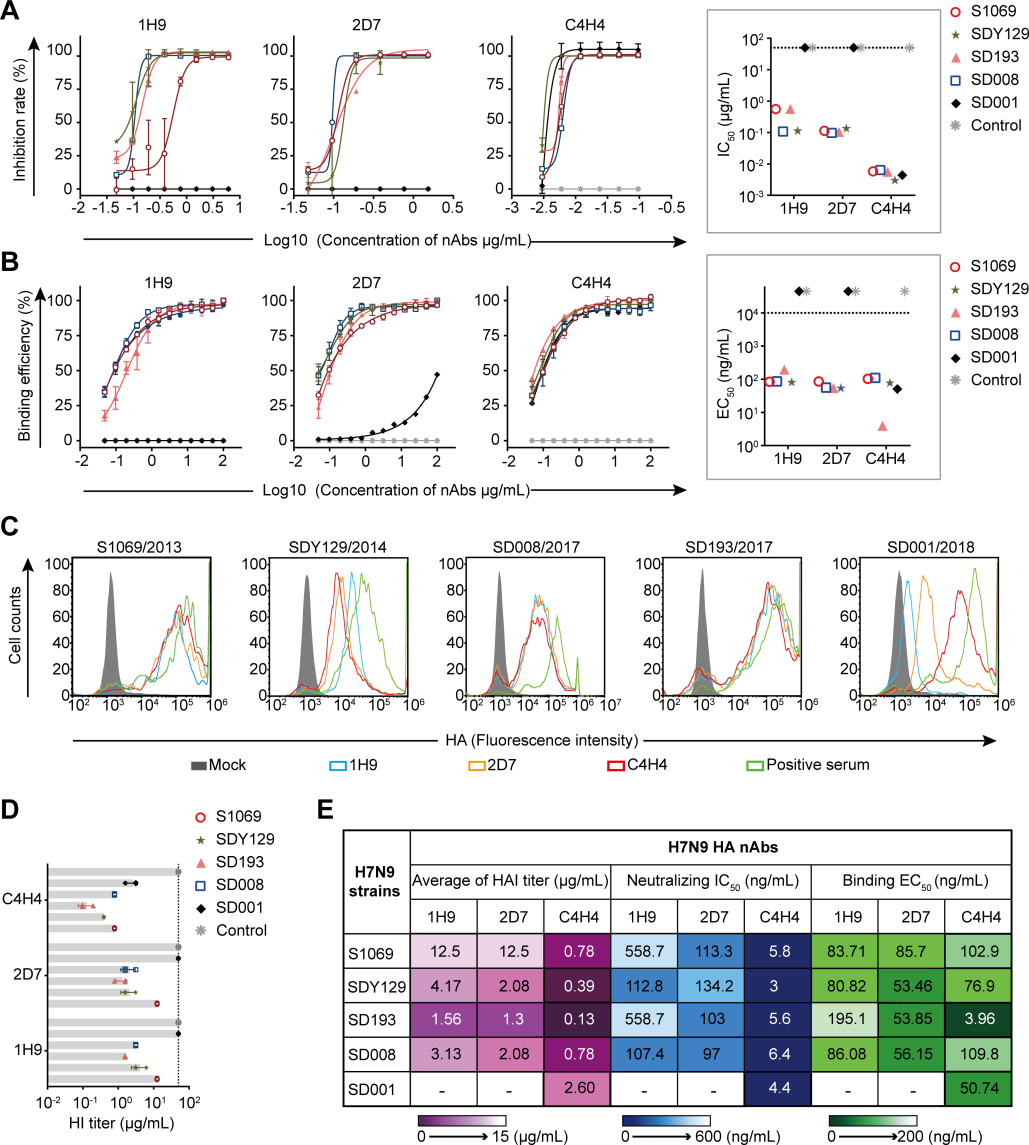
The neutralizing antibodies (nAbs) bind and neutralize H7N9 viruses. (**A**) Inhibition rates of nAbs 1H9, 2D7, and C4H4 against five representative H7N9 strains (S1069, SDY129, SD193, SD008, and SD001) measured by using an microneutralization assay. The half-maximal inhibitory concentration (IC_50_) values of the nAbs were determined from three independent neutralization experiments. IC_50_ values above 50 µg/mL (dashed line) were scored as negative. (**B**) ELISA graphs show the reactivity of nAbs to the viruses. The half-maximal effective concentration (EC_50_) values above 10^4^ ng/mL (dashed line) were scored as negative. (**C**) Flow cytometry plots show the binding capacity of nAbs to the virus-infected cells. MDCK cells were inoculated with H7N9 representative strains. At 24 h post-infection (p.i.), the nAbs that bound to cells were detected with a secondary antibody of wheat germ agglutinin (WGA)-Alexa Fluor 488 conjugate (Thermo Fisher Scientific, Hillsboro, OR, USA) and assessed by flow cytometry. Data are representatives of three repeats. (**D**) Bar graphs show the HI activity of nAbs against H7N9 viruses. HI titers above 50 µg/mL (dashed line) were scored as negative. (**E**) Heatmaps show EC_50_, IC_50_, and HI titers of the nAbs against the viruses. −, negative.

We first assessed the neutralizing activity of these nAbs by using microneutralization (MN) assays and found that C4H4 had strong neutralizing activity against all tested viruses (IC_50_ <10^−2^ µg/mL), whereas 1H9 and 2D7 neutralized all the viruses except for SD001 (Fig. 1A). We then tested the binding affinity of the nAbs to the viruses by using an enzyme-linked immunosorbent assay (ELISA) and found that C4H4 bound efficiently to all the viruses, and the highest affinity was to SD193 (EC_50_ = 3.96 ng/mL), whereas 1H9 and 2D7 failed to bind to SD001 (Fig. 1B). To detect the binding ability of nAbs to H7N9-infected MDCK cells, we sorted the nAbs-bound cells using flow cytometry. The results showed that the nAbs bound to the SD001-infected cells with varying abilities: C4H4 exhibited the strongest binding, 2D7 showed moderate binding, and 1H9 had the weakest binding. For the remaining viruses, all the mAbs demonstrated similar binding abilities (Fig. 1C). We then performed hemagglutination inhibition (HI) assays and found that C4H4 had strong HI activity against all these viruses, whereas 1H9 and 2D7 had no HI activity against SD001 (Fig. 1D). In summary, C4H4 neutralized all five viruses with strong activity. SD001 is an escape strain from 1H9 and 2D7. Additionally, 2D7 and 1H9 show similar neutralizing potency against other viruses (Fig. 1E).

### Broad neutralizing mechanisms of the nAbs

**Fig 2:**
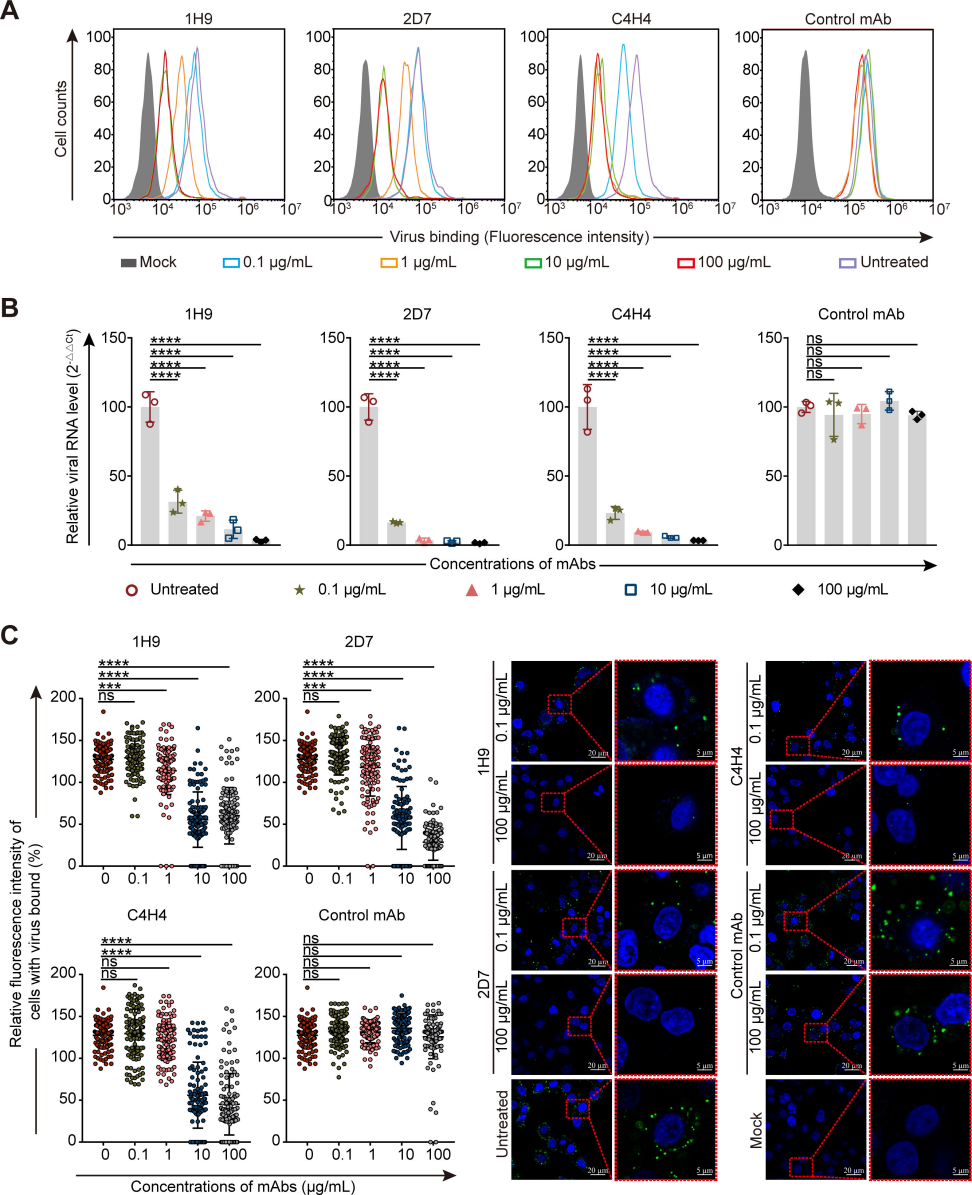
The nAbs inhibit H7N9 viral attachment. (**A**) Flow cytometry plots show the attachment inhibition effect of different concentrations of each nAb to H7N9 strain SD008. (**B**) Bar graphs show the RNA level of the viruses attached to the cell surfaces measured by qPCR. The data show three independent experiments (means ± SDs). (**C**) Viral particles attached to the cell surface were measured by confocal microscopy. The cells were stained with rabbit anti-NP mAb (Sino Biological, Beijing, China) and inoculated with Alexa Fluor 488 highly cross-adsorbed donkey anti-rabbit IgG (H + L) (Thermo Fisher Scientific, Hillsboro, OR, USA). Cell nuclei were stained with 4’,6-diamidino-2-phenylindole (DAPI). The fluorescence intensities of cell-bound virus in at least 110 cells per sample were quantified. A one-way ANOVA with Dunnett’s multiple comparisons test was used for the statistical analysis. ns, not significant, *P < 0.05, **P < 0.01, ***P < 0.001, ****P < 0.0001.

Attachment inhibition is the primary mechanism for nAbs that target the HA receptor binding domain (RBD) (30). To assess the attachment inhibition activity of the nAbs, we performed H7N9 SD008 cell adsorption inhibition experiments. The amount of attached virus was measured by using flow cytometry (Fig. 2A), qPCR (Fig. 2B), and confocal microscopy (Fig. 2C). We found that these nAbs inhibited SD008 attachment to A549 cells in a concentration-dependent manner. A concentration as low as 10 µg/mL of each nAb significantly inhibited attachment.

**Fig 3:**
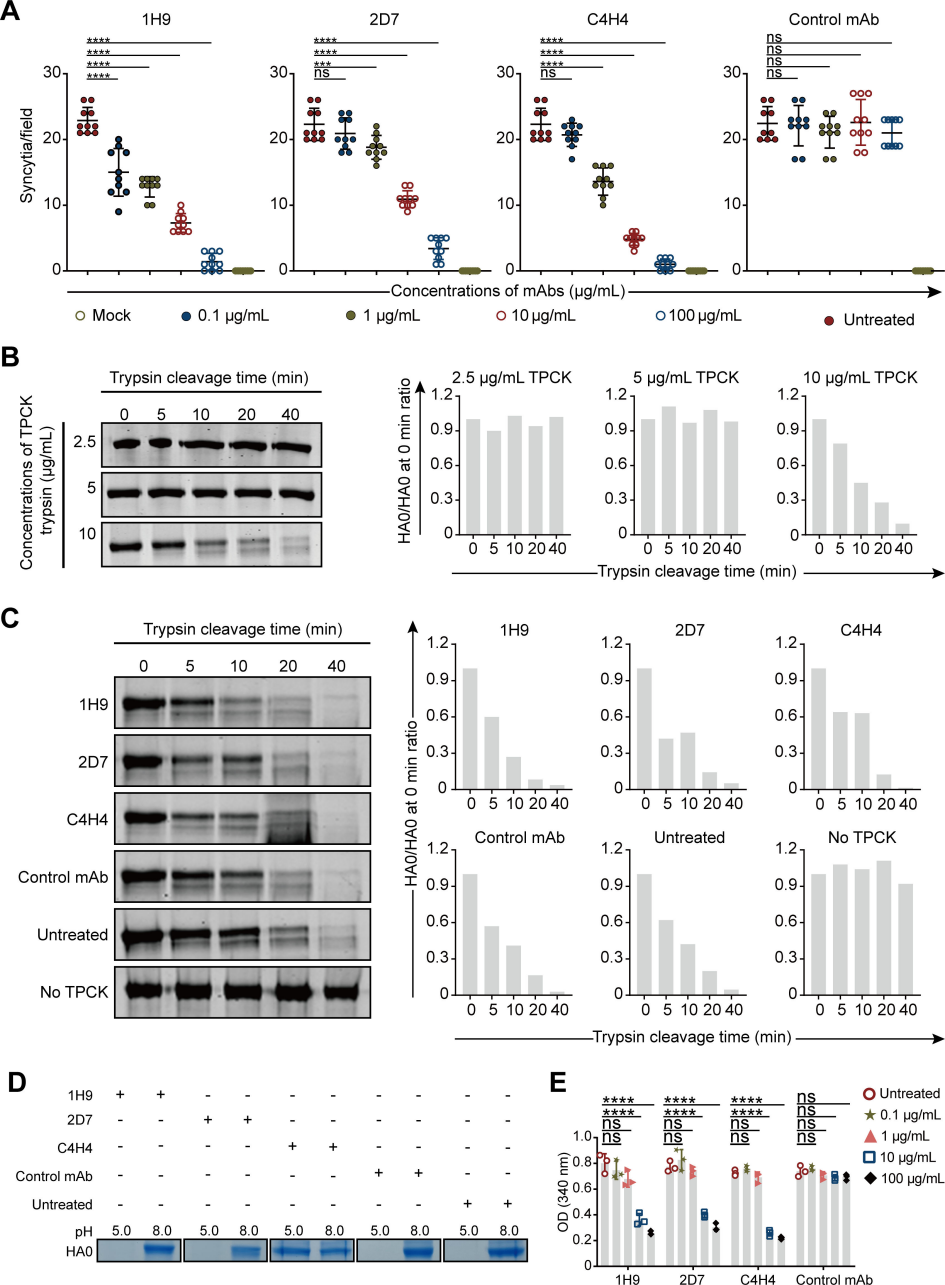
The nAbs inhibit H7N9 viral membrane fusion. (**A**) Scatterplots show the average numbers of syncytia induced by SD008. The data show 10 independent fields, imaged at 20× magnification to count (means ± SDs). (**B**) SDS-PAGE and bar graphs show the cleavage of HA by trypsin at different concentrations. The band intensities of the SDS-PAGE were quantified and are indicated by relative ratios of HA0/HA0 at 0 min. (**C**) SDS-PAGE and bar graphs show the nAb inhibition of HA cleavage by trypsin with the TPCK-trypsin concentration determined in panel **B**. (**D**) Nonreducing SDS-PAGE shows the inhibitory effect of the nAbs on the HA conformational change induced by low pH (pH = 5.0). Data presented are representative experiments from three independent experiments. (**E**) Bar graph shows the amount of NADPH released into the suspension to indicate the inhibitory activity of the nAbs against the fusion-induced red blood cell (RBC) lysis. The data show three independent experiments (means ± SDs). A one-way ANOVA with Dunnett’s multiple comparisons test was used for the statistical analysis. ns, not significant, *P < 0.05, **P < 0.01, ***P < 0.001, ****P < 0.0001.

To assess whether the three nAbs inhibit the membrane fusion process, we performed syncytium formation inhibition assays and found that the number of virus-induced syncytia in A549 cells decreased gradually as the concentration of each nAb increased. The concentrations that resulted in a significant decrease in syncytia were ≥0.1 µg/mL, ≥1 µg/mL, and ≥1 µg/mL for 1H9, 2D7, and C4H4, respectively (Fig. 3A; Fig. S2), indicating that these nAbs efficiently inhibit virus-induced membrane fusion. To achieve membrane fusion, the HA of influenza virus must be firstly cleaved into HA1 and HA2 by host cell proteases (31). We performed trypsin HA cleavage assays and found that the nAbs did not inhibit the cleavage of recombinant HA by trypsin (Fig. 3B and C). After being cleaved, the conformation change of HA at low pH is necessary to expose the fusion peptide for membrane fusion (11). We then conducted HA conformational change inhibition analyses and found that only C4H4 inhibited low pH-induced (pH = 5.0) HA1 disassociation (Fig. 3D). Further analysis showed that each nAb at a concentration of >10 µg/mL significantly inhibited fusion peptide-induced cell membrane disruption (Fig. 3E). Collectively, these data demonstrate that the nAbs inhibit viral membrane fusion by perturbing fusion peptide-mediated cell membrane disruption and that C4H4 also inhibits the conformational change of HA.

**Fig 4:**
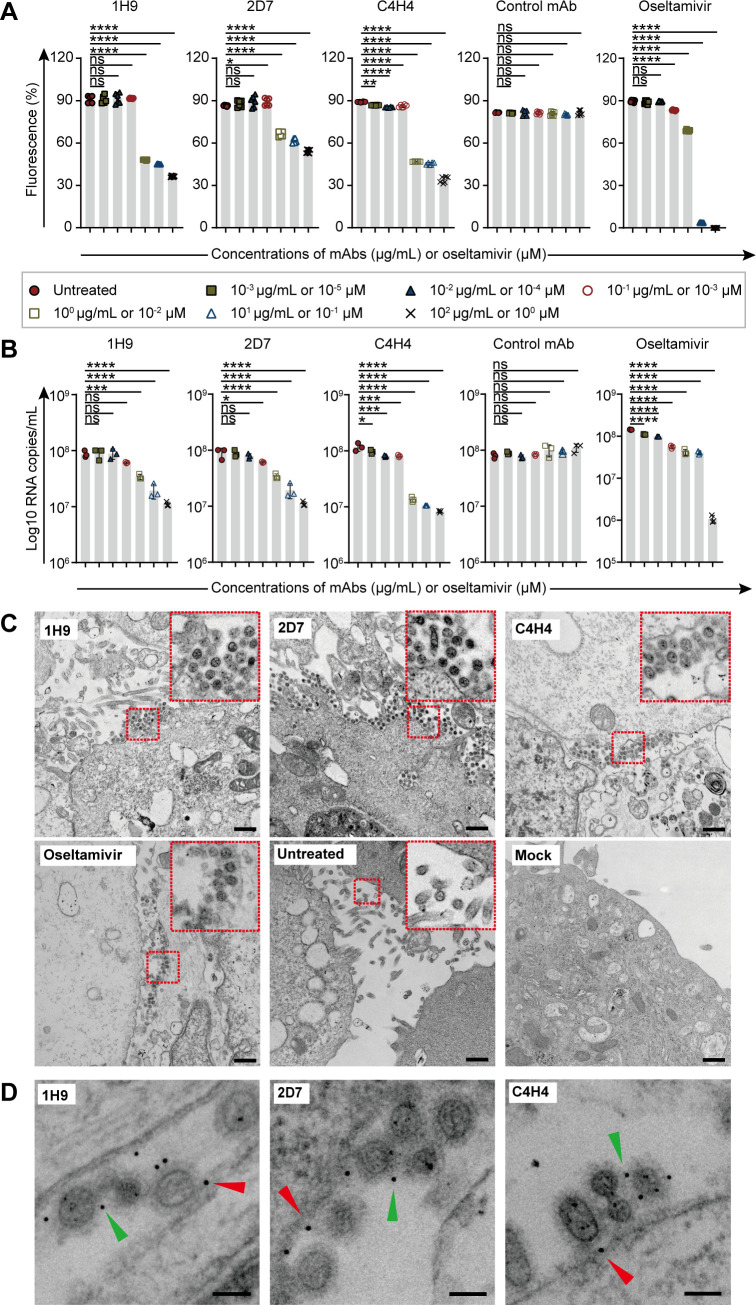
The nAbs inhibit H7N9 viral egress and release. (**A**) Bar graphs show the fluorescence intensity from the free viruses in the supernatant of MDCK cell cultures measured by reading fluorescence by using a Victor V plate reader (Perkin Elmer) with the following settings: excitation 355 nm; emission 460 nm; and 10 flashes per well. The data are the means ± SD of six experiments. (**B**) Bar graphs show the RNA copy number of the viruses in the supernatant of MDCK cell cultures measured by qPCR. The data show three independent experiments (means ± SDs). (**C**) Electron micrographs of H7N9-infected MDCK cells show the distributions of viruses after being treated with 1H9, 2D7, C4H4, a control mAb, or oseltamivir. The scale bar is 1 µm. The insets are the enlarged view of the field in the red frames. (**D**) Immunogold labeling images show the binding of nAbs to viral particles and the HA expressed on the cell surface. The second antibody is a goat anti-mouse IgG conjugated to 10 nm gold particles. The scale bar is 100 nm. The red triangles point to the nAbs that bound to the HAs from a virus and the cell surface, whereas the green triangles point to the nAbs that bound to the HAs from two neighboring viral particles. A one-way ANOVA with Dunnett’s multiple comparisons test was used for the statistical analysis. ns, not significant, *P < 0.05, **P < 0.01, ***P < 0.001, ****P < 0.0001.

Some HA-targeted nAbs also inhibit viral egress and release through Fc-mediated steric hindrance to the NA active site (32, 33). We assessed our nAbs for inhibition of virus egress and release in MDCK cells by using a methyl-umbelliferyl neuraminic acid (MU-NANA) cleavage assay, qPCR, and electron microscopy (EM). Oseltamivir-treated cells were used as a positive control. In our qPCR assay, we employed absolute quantitative real-time PCR to measure vRNA copies in the cell culture medium. This method provided an indirect quantification of released viral particles. In the MU-NANA cleavage and qPCR assays, both the fluorescence intensity and the amount of vRNA in the supernatants decreased as the nAb concentration increased. A concentration as low as 1 µg/mL of each nAb induced a significant decrease in these measurements (Fig. 4A and B). The EM micrographs showed that a few viruses were distributed randomly between the intercellular spaces in the control cells, whereas in the oseltamivir- and nAb-treated cells, progeny viral particles were retained on the surface of infected cells (Fig. 4C). Gold labeling analysis corroborated that the nAbs induced this retainment of the virus on the infected cells’ surface (Fig. 4D). These results indicate that the three nAbs significantly inhibit H7N9 virus release by retaining progeny viruses on the infected cell surface, thereby preventing further infection of neighboring cells.

Furthermore, we observed viral particles retained on the cell surface being invaginated by cellular processes, with large vesicles containing numerous viral particles in the cytosol (Fig. S3A and B). Further examination with immunogold EM revealed that these viral particles in the vesicles were taken up from outside the cells (Fig. S3C). We also found some vesicles containing lysosomes and small empty virus-like vesicles (Fig. S3D). These results suggest that the unsuccessfully released progeny viruses may be taken up again by the infected cell through macropinocytosis and degraded through the lysosomal pathway. However, the exact mechanism needs further exploration in future studies.

### Cryo-EM structures of HA trimer and HA-Fab complexes

To explore the structural basis of the neutralizing capacity of these nAbs, we determined the cryo-EM structures of the SD008 HA trimer alone and in complex with the Fab of each nAb.

**Fig 5:**
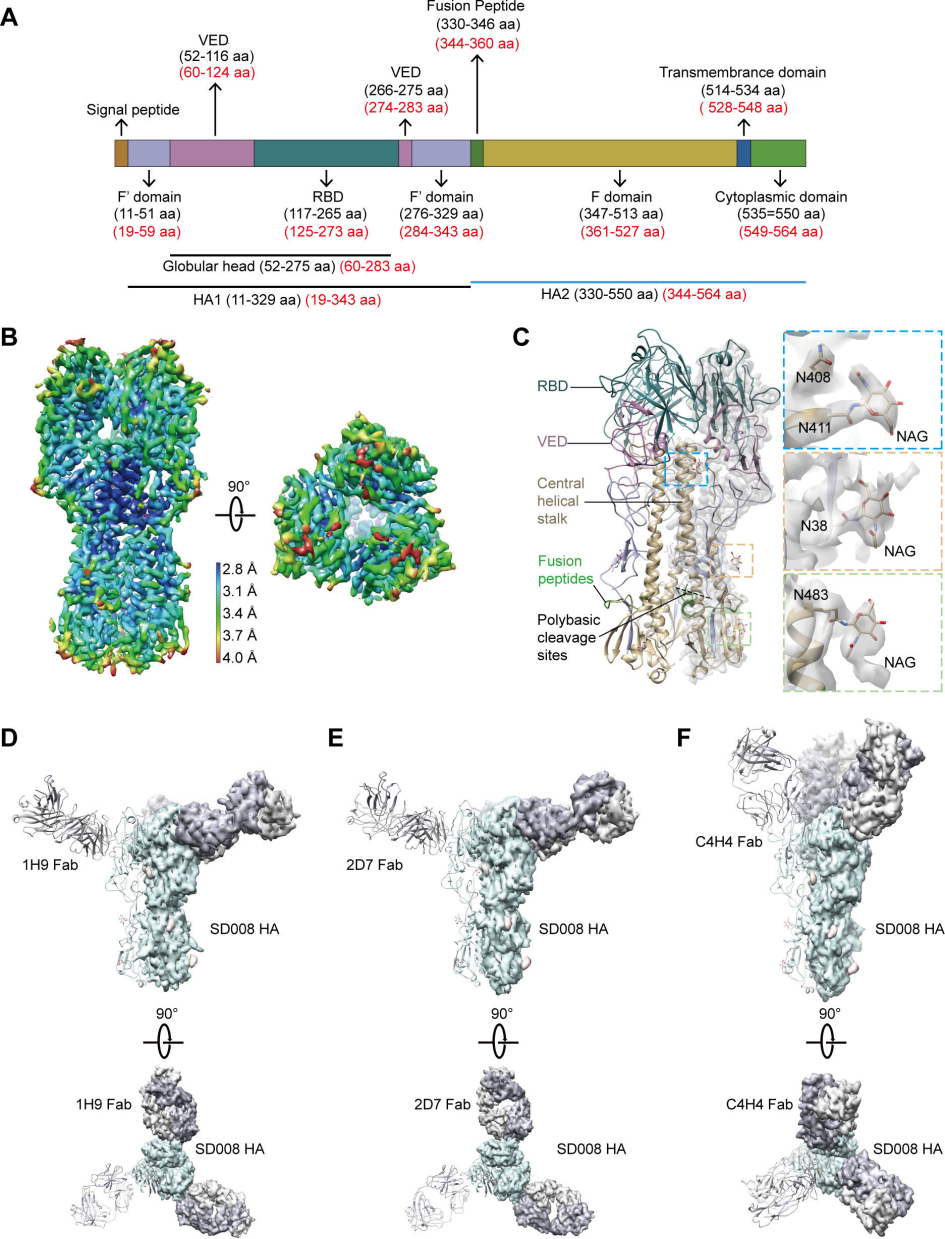
Cryo-electron microscopy (cryo-EM) maps of the SD008 HA trimer and HA-Fab complexes. (**A**) Schematic view of SD008 HA 2D structure. The structure starts from its N-terminal signal peptide (10 aa), and the stalk domain is divided into the F’ (purple) and F (yellow) subdomains, whereas the globular head consists of the receptor binding domain (RBD) (dark green) and vestigial esterase domain (VED) (dark pink), followed by the fusion peptide (green), transmembrane domain (blue), and cytoplasmic domain (light green). Numbering is indicated in the H3 (black) and SD008 H7 (red) formats. (**B**) The side and top views of the EM map of the SD008 HA trimer are shown. The map is colored according to the local resolutions estimated with the gold standard Fourier shell correlation (FSC) criterion of 0.143. (**C**) Ribbon representation of the SD008 HA trimer with one promoter fitted into its corresponding electron microscopy (EM) map. Three N-linked glycans were found at sites N38 (orange), N411 (blue), and N483 (green). The RBD, VED, central helical stalk, fusion epitope, and polybasic cleavage sites are labeled and colored with the corresponding colors of the schematic map shown in panel **A**. The side and top views of the structures of HA-1H9 Fab (**D**), HA-2D7 Fab (**E**), and HA-C4H4 Fab (**F**) fit with the corresponding EM maps. A promoter in each complex is ribbon presented. The HA, antigen-binding fragments (Fabs), and glycans are colored light green, gray, and pink, respectively.

To determine the cryo-EM structure of the SD008 HA trimer, we collected 61,557 particles from 623 movies and obtained a map with an overall resolution of 3.1 Å at a 0.143 Fourier shell correlation (FSC), which we then used to build the atomic model of the HA (Fig. S4A , Table S1). The structure of the SD008 HA was congruent with the corresponding moiety of the NY107, NL219, and SH-2 HA structures (PDB code: 3M5G, 4DJ6, and 4LN6), as manifested by a structural superimposition of a root mean square deviation (RMSD) of 1.006, 0.946, and 1.037 Å for all HA C-α atoms (Fig. S5) (34–36). All subdomains of the HA are clearly defined except for the polybasic cleavage sites, which are missing from the density probably due to the high flexibility of the local structure (Fig. 5A through C). Interestingly, we identified three N-linked glycans at positions N38, N411, and N483 (Fig. 5C). Sequence analysis showed that these sites are completely conserved among all available H7N9 strains, suggesting that these glycosylation modifications may be important to the biological characteristics of H7N9 viruses.

Our initial HA-Fab complex reconstructions suggested that the complexes adopted preferred orientations (preferred “top” orientation, but lacking tilted side views). This problem has also been reported in other structural studies of the influenza HA trimer and studies of the SARS-CoV S trimer (19, 37–39). We overcame the problem by adopting the recently developed strategy of collecting an additional data set at a 40° tilt angle (40). After combining the data collected at 0° and 40° tilt angles, we performed three-dimensional reconstruction and obtained cryo-EM maps of HA complexed with the Fab of 1H9, 2D7, and C4H4 at resolutions of 2.9 Å, 3.0 Å, and 2.9 Å, respectively, by using the FSC = 0.143 criterion (Fig. S4B through D). The atomic models of the three complexes were then built by fitting the obtained HA structure and the homology models of the three Fabs into the corresponding densities and refined in Coot (41). In the HA-Fab complexes, the densities that are responsible for the HA trimer and the Fab of the nAbs are clearly defined, revealing that all the Fabs bind to the head domain of HA but target different sites. The 1H9 and 2D7 engaged sites are located on the side of the HA head and their Fabs point nearly horizontally from the side of each HA promoter, whereas C4H4 binds to a site on the top of HA and its Fabs extend obliquely upward from the HA head (Fig. 5D through F).

### Interactions between HA and the Fab of 1H9 and 2D7

**Fig 6:**
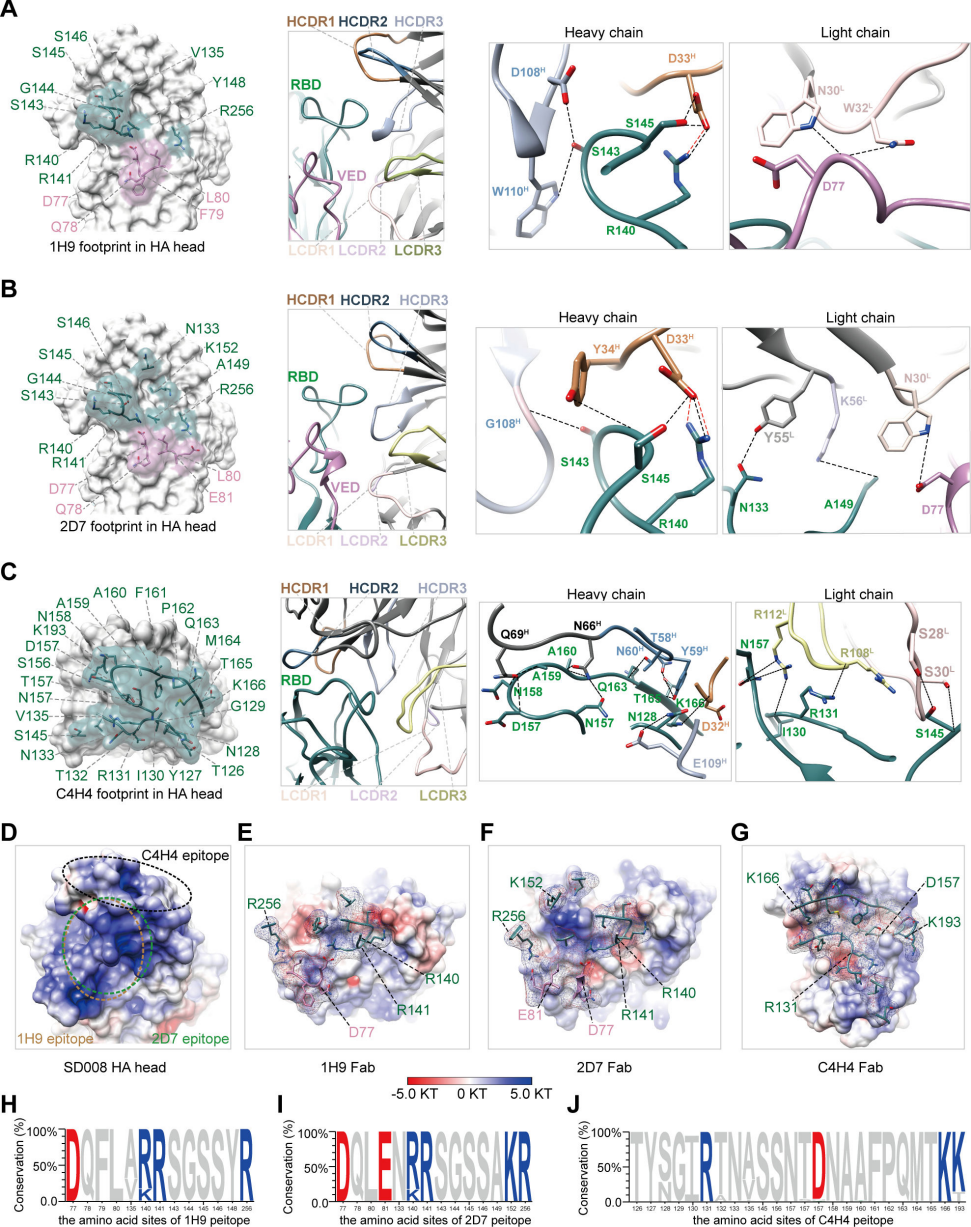
Interactions between HA and the Fabs of 1H9 (**A**), 2D7 (**B**), and C4H4 (**C**). (**A-C**) Graphs from left to right show the footprints of the Fab with the residues labeled and displayed as sticks, the loops involved in the interface of the complex, and hydrogen bond interactions formed between HA and the H and L chains of the Fab, respectively. Residues from RBD and VED are colored green and purple, respectively. Hydrogen bonds and salt bridges are shown as black- and red-dashed lines, respectively. (**D-G**) Electrostatic potential surfaces of the epitopes (**D**) and the Fabs of 1H9 (**E**), 2D7 (**F**), and C4H4 (**G**). The dashed circles in (**D**) indicate the locations of the epitopes of 1H9 (orange), 2D7 (green), and C4H4 (black). The electrostatic potential surface is colored with gradient color: red, negative, −5KT; blue, positive, +5 KT; and white, neutral. The epitope amino acid residues involved in the interaction with the Fab are displayed as sticks, and polar residues are labeled. (**H-J**) Entropy plots show the variation of the epitope amino acids for 1H9 (**H**), 2D7 (**I**), and C4H4 (**J**). Amino acids colored red are negatively charged, those colored blue are positively charged, and those in gray are neutral.

The most parts of 1H9 and 2D7 Fab binding sites are overlapped and span the RBD and vestigial esterase domain (VED) (Fig. 6A and B). The buried surface area (BSA) of the 1H9 heavy and light chains are 343.6 Å^2^ and 373.8 Å^2^, respectively (Table S2; Table S2). According to the van der Waals radii (< 4.0 Å), the footprint of the 1H9 Fab involves 13 amino acid residues, nine of which are in the RBD, whereas the other four belong to the VED (Fig. 6A; Table S3). To engage the 1H9 Fab, the 130-loop of HA protrudes into the canyon formed by the heavy chain complementarity-determining region (HCDR)−2 and HCDR3 of the 1H9-Fab, forming a spatially favorable position for binding (Fig. 6A). Seven hydrogen bonds and one salt bridge are formed between the residues R140, S143, S145, and D77 of HA and D33^H^, D108^H^, W110^H^, N30^L^, and W32^L^ from 1H9 Fab (Fig. 6A; Table S2). The BSAs of the H and L chains of the 2D7 Fab are 367.8 Å^2^ and 359.0 Å^2^, respectively (Table S4). The footprint of 2D7 consists of 14 amino acids (Fig. 6B; Table S5). There are 10 shared amino acids between the epitope of 1H9 and 2D7 (Fig. 6A and B; Table S3 and S5). In the 2D7 Fab-HA complex, two additional hydrogen bonds are formed between the residues HA N133 and A149 and Fab Y55^L^ and K56^L^, suggesting that 2D7 has a stronger binding affinity for HA than that of 1H9 (Fig. 6B; Table S4).

### Interactions between HA and C4H4 Fab

The C4H4 binding site is located on the top of HA, and the BSAs defined by the H and L chains are 770.1 Å^2^ and 451.8 Å^2^, respectively (Fig. 6C; Table S6). The C4H4 epitope consists of 24 amino acids mainly from the 130- and 150-loop, which interact extensively with residues from the HCDRs and LCDRs of the C4H4 Fab (Fig. 6C; Table S7). Hydrogen binding contributes to most of the interactions between the HA and the Fab, involving 18 hydrogen bonds (Fig. 6C; Table S6).

We further analyzed the surface electrostatic potential of the interface of the complexes and found that the epitope surfaces are positively charged. In contrast, the corresponding binding surfaces of the Fabs are largely negatively charged, suggesting that electrostatic potential also contributes to the engagement of the nAbs with HA (Fig. 6D through G). The epitope of 2D7 has two more polar amino acids (negatively charged E81 and positively charged K152) and is, therefore, more polar than that of 1H9. Based on an analysis of 1,305 HA amino acid sequences, most of the amino acids in the three epitopes are highly conserved (>90%), suggesting that these nAbs can probably neutralize most H7N9 viruses (Fig. 6H through J).

### G144E mutation in HA leads to SD001 neutralization escape

**Fig 7:**
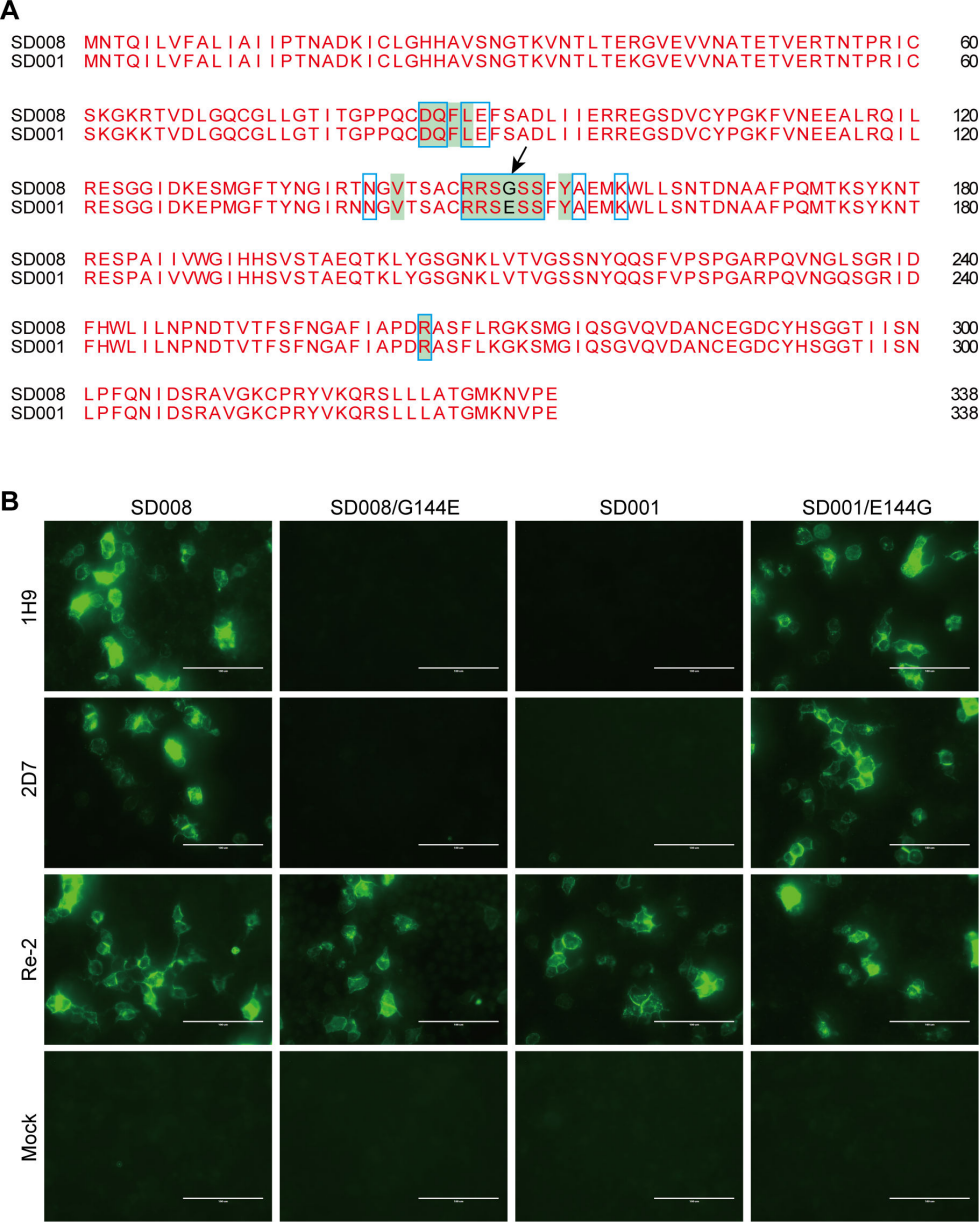
G144E mutation in HA leads to SD001 neutralization escape. (**A**) The HA/G144E mutation contributes to the neutralization escape of SD001 from 1H9 and 2D7. Sequence alignment shows the HA/G144E mutation (pointing arrow) presented in SD001. The amino acid sites of the 2D7 epitope are marked with a blue wireframe, whereas those of 1H9 are shown in green in the background. (**B**) IFA results show that the HA/G144E mutation leads to the neutralization escape of SD001 from 1H9 and 2D7. HEK293T cells were transfected with the plasmids of pCDNA3.1-SD008-HA, pCDNA3.1-SD001-HA, pCDNA3.1-SD008-HA/G144E, or pCDNA3.1-SD001-HA/E144G for 24 h, stained with 1H9, 2D7, and a chicken-derived serum and the mAbs Alexa Fluor 488 highly cross-adsorbed donkey anti-mouse IgG or goat anti-chicken IgY (H + L) (Thermo Fisher Scientific, Hillsboro, OR, USA), respectively, and visualized on an inverted fluorescence microscope (EVOS M5000 Cell Imaging System, Life Technologies). Scale bar represents 100 µm.

Given that the two nAbs, 1H9 and 2D7, exhibit robust neutralizing activities against H7N9 viruses such as SD008 but not SD001, we compared the amino acid differences in their HA epitopes (Fig. 7A). We found a unique G144E mutation in the HA of SD001, which is likely responsible for its escape from neutralization (Fig. 7A). We then investigated the impact of the HA G144E mutation on SD001 neutralization escape. We constructed the plasmids of pcDNA 3.1-SD008-HA, pcDNA 3.1-SD008-HA/G144E, pcDNA 3.1-SD001-HA, and pcDNA 3.1-SD001-HA/E144G. These expressed SD008 HA, SD008 HA with a G144E substitution, SD001 HA, and SD001 HA with a E144G mutation, respectively. The immunoreactivity of the expressed proteins was tested with the two nAbs in HEK293T cells using an immunofluorescence assay. As expected, the SD008 HA with the G144E mutation lost reactivity with the two nAbs. Conversely, the SD001 HA with the E144G mutation demonstrated robust reactivity with the two nAbs (Fig. 7B). These results provide concrete evidence that the G144E mutation in the HA of SD001 leads to neutralization escape.

**Fig 8:**
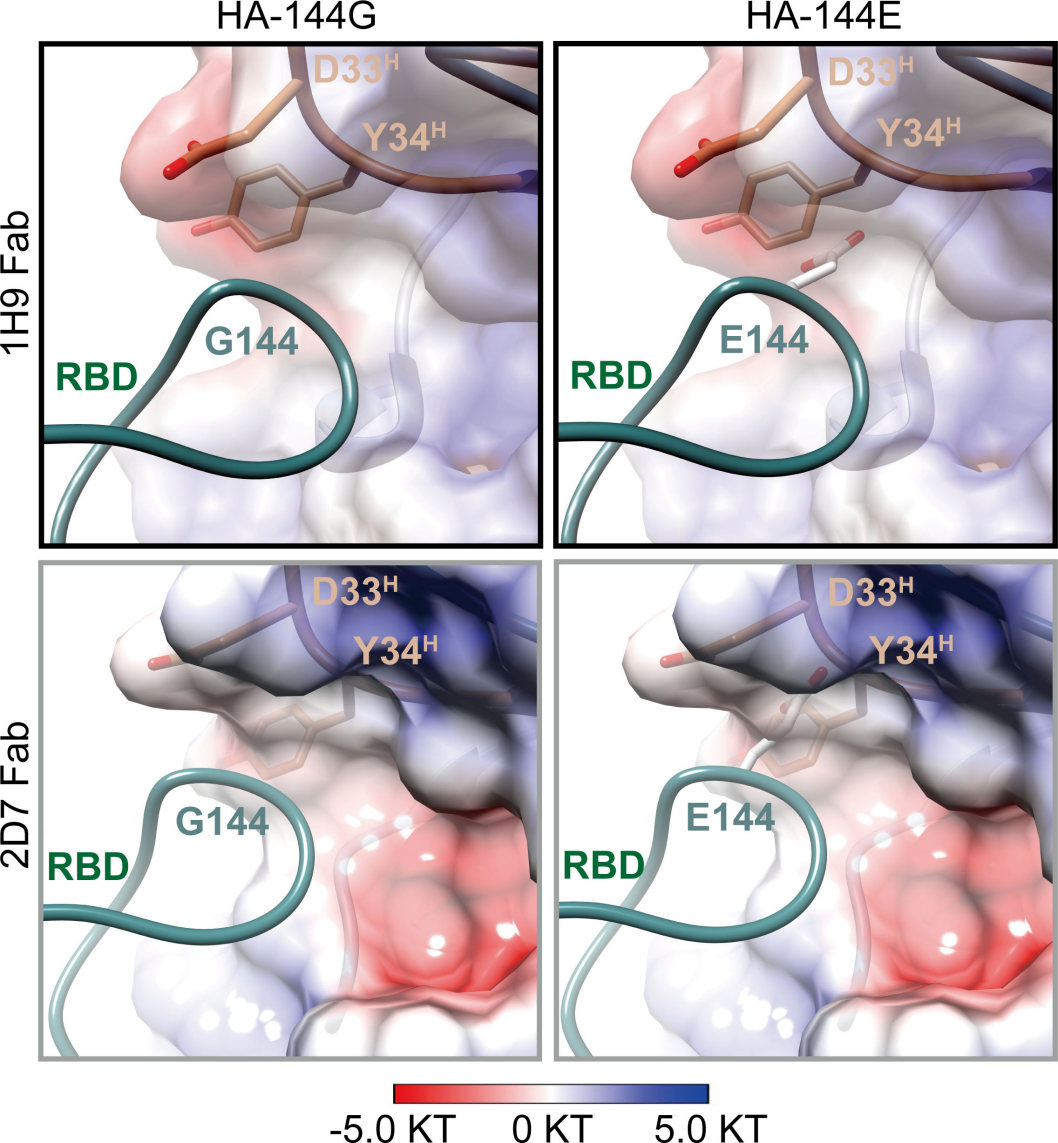
Structural basis for the escape of SD001 from neutralization by 1H9 and 2D7. Structural analyses reveal the bulk side chain of E144 creates a steric hindrance for the nAbs 1H9 and 2D7 in engaging the HA of SD001. The electrostatic potential surfaces of 1H9 and 2D7 are shown in gradient colors: red, negative, −5KT; blue, positive, +5 KT; white, neutral.

To understand the neutralization escape by SD001, we introduced the G144E mutation into the HA of the cryo-EM structures. These were then optimized via energy minimization. We found that the HA/G144E mutation causes clear steric hindrance for the Fabs in engaging the HA. The bulkier side chain of Glu overlaps with the side chain of Y34^H^. Furthermore, Glu is acidic and negatively charged, creating a repulsive force against the local binding surface (Fig. 8). These results suggest that E144 in the HA of SD001 obstructs the binding of 1H9 and 2D7 nAbs through multiple mechanisms.

### Structural impact of R140K mutation on HA-C4H4 binding

**Fig 9:**
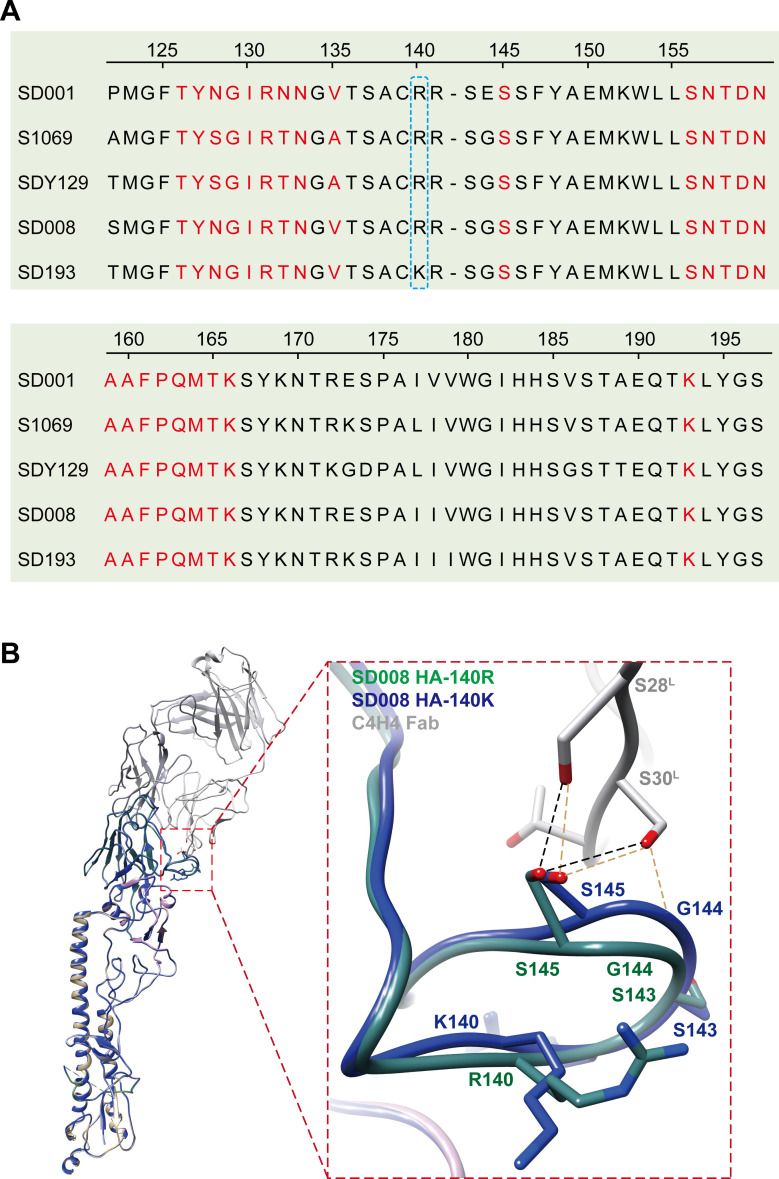
Structural basis for the impact of R140K mutation on HA-C4H4 binding. (**A**) The R140K mutation in the HA of SD193. The panel shows that R140K mutation occurred in the HA of SD193 (blue-dashed line box), and the amino acid sites of the C4H4 epitope are colored red. (**B**) The panel shows the structural simulation results of the R140K mutation based on the SD008 HA. Black-dashed lines, hydrogen bonds formed between C4H4 antigen-binding fragment (Fab) and SD008 HA-140R; orange-dashed lines, hydrogen bonds between C4H4 Fab and SD008 HA-140K.

Our study found that C4H4 has a stronger binding affinity to SD193 (Fig. 1B). To explain this phenomenon, we compared the amino acid differences in C4H4 epitopes in the five representative viruses (Fig. 9A). We found a unique R140K mutation in the HA of SD193, which is likely responsible for its strong binding affinity to C4H4. The R140K mutation is located in the 130-loop, the core component of the C4H4 epitope (Fig. 9A). To understand the strong binding affinity of SD193 to C4H4, we introduced the R140K mutation into the HA of the cryo-EM structures. These were then optimized via energy minimization. We found that the HA/R140K mutation in the 130-loop pushes the up edge of the 130-loop closer to the L chain of the Fab, which leads to the formation of an additional hydrogen bond between HA G144 and C4H4 S30^L^ (Fig. 9B). These results suggest that K140 in the HA of SD193 enhanced its binding affinity to C4H4 through shortening the distance between HA and L chain of C4H4.

### Therapeutic efficacy of C2D7 and CC4H4 against H7N9 infection in mice

Many nAb-based therapeutics are effective in treating virus infections (12, 42, 43). Given that the 1H9 and 2D7 epitopes largely overlap and that 2D7 has a higher binding ability than 1H9, we developed humanized chimeric nAbs C2D7 and CC4H4 and evaluated their potential as H7N9 therapeutics. A dose of 25 MLD_50_ (the 50% mouse lethal dose) of the H7N9 strain A/chicken/Guangdong/SD008/2017-PB2/627K (CK/SD008-PB2/627K), which is highly lethal in mice, was used to infect the mice.

**Fig 10:**
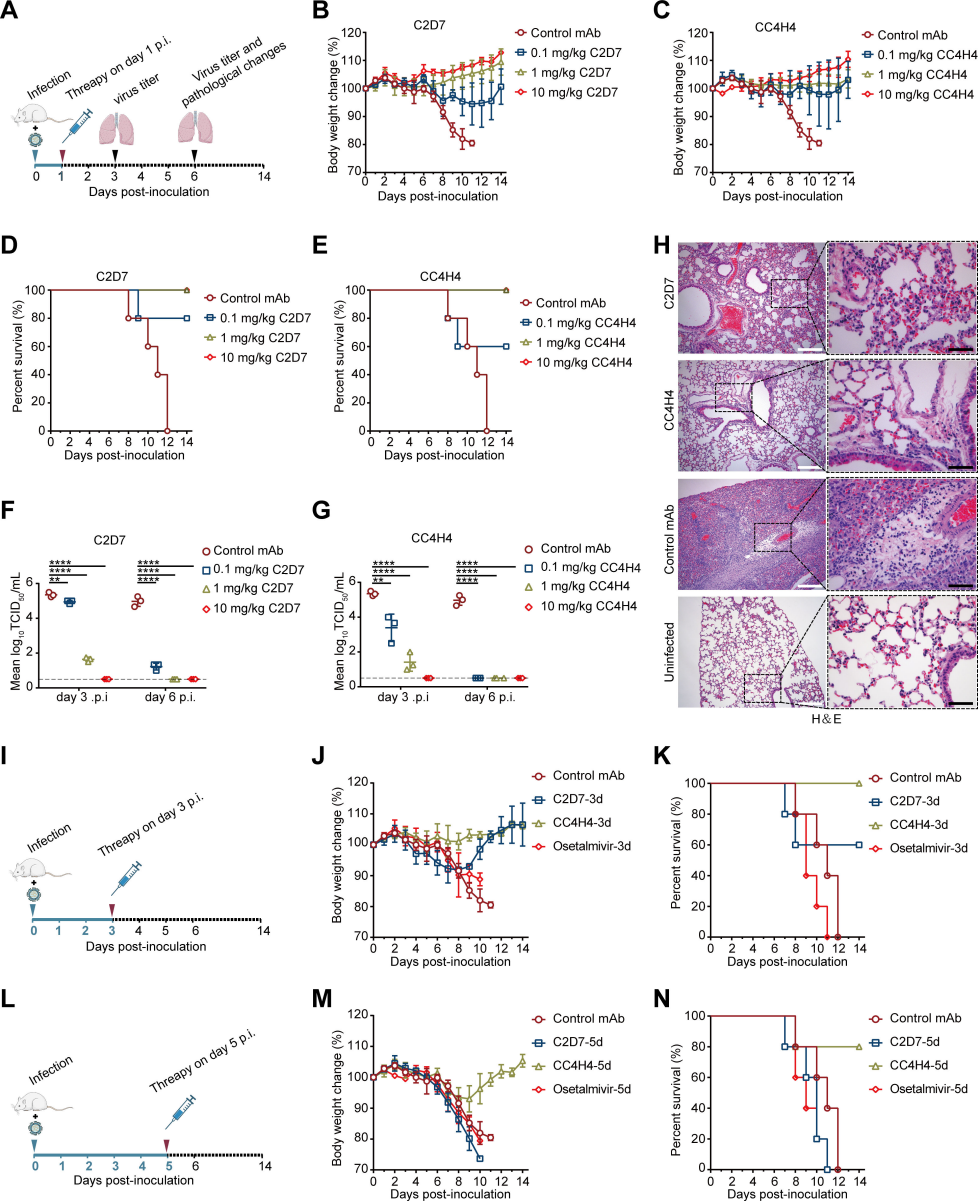
The therapeutic effect of nAbs on infection with H7N9 virus in mice. (**A**) Flow chart of treatment of H7N9 infection in mice with the nAbs. The changes in body weight (**B and C**), survival rates (**D and E**), and virus loading in the lungs (**F and G**) in BALB/c mice (*n* = 11 or 14 per group) infected with 25 MLD_50_ of an H7N9 strain (SD008) and then treated intravenously with humanized C2D7 (0.1, 1, and 10 mg/kg), CC4H4 (0.1, 1, and 10 mg/kg), or a control mAb (10 mg/kg) on day 1 p.i., respectively. A one-way ANOVA with Dunnett’s multiple comparisons test was used to determine significant differences in virus titers in the lungs compared with the control mAb-treated group. *P < 0.05, **P < 0.01, ***P < 0.001, ****P < 0.0001. The dashed lines show the lower limit for virus detection. (**H**) Lung damage in the mice treated with C2D7 (10 mg/kg), CC4H4 (10 mg/kg), or a control mAb (10 mg/kg) on day 6 p.i. was detected by hematoxylin-eosin (H&E) staining. The white and black scale bars represent 200 and 50 µm, respectively. (**I-N**) The effect of delayed treatment of H7N9 infection in mice with the nAbs. Flow charts of delayed treatments of H7N9 infection in mice with the nAbs or oseltamivir on days 3 (**I**) and 5 (**L**) p.i.. Changes in body weight (**J and M**) and survival rate (**K and N**) of mice (*n* = 5 per group) infected with 25 MLD_50_ of the H7N9 virus and then intravenously administered 10 mg/kg C2D7 or CC4H4, or orally administered 25 mg/kg oseltamivir on days 3 or 5 p.i., respectively.

To assess the therapeutic effect, the infected mice were treated with three doses (0.1 mg/kg, 1 mg/kg, or 10 mg/kg) of C2D7, CC4H4, or an unrelated monoclonal antibody (mAb) on day 1 post-infection (p.i.), and body weight, survival rate, tissue virus loading, and pathogenic lesions in the lungs were monitored (Fig. 10A). We found that all mice treated with 1 or 10 mg/kg of either of the two nAbs survived without obvious weight loss, whereas the controls showed a rapid decrease in body weight and died within 12 days of infection (Fig. 10B through E). On day 3 or 6 p.i., the lung viral titers in the nAb-treated mice were significantly lower than those in the control mice (Fig. 10F and G). The lungs of the control mice showed severe infiltration of fibrin and inflammatory cells in the alveoli and bronchiole; the lungs of the C2D7-treated mice exhibited mildly widened alveolar-septa due to proliferation of pneumocytes and papillary congestion; of note, the lungs of CC4H4-treated mice appeared nearly normal (Fig. 10H). Collectively, these results indicate that both nAbs provide therapeutic protection against H7N9 infection in mice.

Furthermore, to assess the therapeutic effect of delayed treatment with the nAbs, infected mice were treated with 10 mg/kg of either of the two nAbs or 25 mg/kg oseltamivir on day 3 or 5 p.i. (Fig. 10I and L). Oseltamivir has proven to be less effective against influenza virus infection when its administration is delayed (44, 45). As expected, all the mice that were treated with oseltamivir on day 3 or 5 p.i. experienced severe body weight loss and died by day 11 p.i.. In the group treated with C2D7 on day 3 p.i., 60% of the mice survived, and their body weight recovered rapidly after day 8 p.i.; however, all mice died by day 11 p.i. in the group treated with C2D7 on day 5 p.i.. Surprisingly, 100% of the mice treated with CC4H4 on day 3 p.i. survived without notable weight loss, and 80% of the mice survived in the group treated with CC4H4 on day 5 p.i., suggesting that C4H4 has therapeutic potential against H7N9 infection (Fig. 10J through N).

## DISCUSSION

H7N9 viruses have triggered significant global health concerns due to the severe infection they cause in humans and their continuous antigenic evolution (46). Although human infections have stopped since the vaccination of chickens in China, H7N9 viruses have not been eradicated from poultry populations (28, 29). The 627K and 701N mutations in PB2 gene and 156D mutation in M1 gene make it easier for H7N9 viruses to infect mammalian hosts, which highlights the need for the development of effective vaccines and drugs to fight against potential new outbreaks (47, 48). In this study, we developed three nAbs (1H9, 2D7, and C4H4) against H7N9 viruses and found that they neutralized the virus by inhibiting viral attachment, membrane fusion, and egress and release but did so with different capacities. We then resolved the cryo-EM structures of HA in complex with the Fab of each nAb and found the molecular determinants of their binding activities to HA. Finally, we proved that humanized chimeric nAbs C2D7 and CC4H4 were effective in treating mice infected with lethal H7N9 virus.

H7N9 viruses have undergone continuous evolution, and the amino acid mutations in the HA protein of some variant strains have enabled them to circumvent neutralization by antibodies (49). To test the neutralizing capacity of the three nAbs, we selected five representative H7N9 viruses that belong to different evolutionary branches. C4H4 neutralized all these viruses with high activity. Its epitope consists of 24 amino acids, which extensively interact with residues from the Fab mainly by hydrogen bonding, explaining its broad neutralizing capacity. Consistent with other studies, nAbs engaging similar epitopes showed a broad neutralizing spectrum against H1N1 and H5N1 viruses (50, 51). Interestingly, C4H4 showed the strongest binding affinity to SD193, which bears a R140K mutation in HA, pushes the up edge of the 130-loop closer to the L chain of the Fab, and leads to the formation of an additional hydrogen bond between HA G144 and C4H4 S30^L^. The SD001 strain has a distinct G144E mutation compared with other viruses. Glu is negatively charged and has a bulky side chain, which created a repulsive force and steric hindrance with Y34^H^ of the 1H9 and 2D7 Fabs (interatomic distance <1 Å). Together, our findings provide new insight into the neutralization and escape mechanisms of H7N9 viruses.

HA-targeted nAbs neutralize influenza viruses through multiple mechanisms, such as blocking receptor binding (52, 53), inhibiting viral and cellular membrane fusion (16, 54), and preventing egress and release of progeny virions (13). In this study, we found that the epitopes of the three nAbs are all located on the HA head. Therefore, it is not surprising that they block H7N9 virus attachment. However, we also found that they inhibited low pH-induced membrane fusion, which has only been found in nAbs that target epitopes near the cleavage site of HA (16–18, 55). We found that these three nAbs did not influence HA cleavage but inhibited fusion peptide-mediated membrane disruption, and only C4H4 prevented the conformational change of the HA. The engagement of the nAbs with HA may result in steric hindrance and interfere with the disruption of the membrane. Previous studies have shown that nAbs bound to a similar epitope as that of C4H4 inhibit the conformational change of HA (51, 56), but the underlying mechanism remains unknown. Our three nAbs inhibited the viral egress and release by linking the viruses with the membrane, forming stable connections to prevent the release of the viral particles. This nAb-binding-induced release inhibition and re-entry of progeny viral particles may play an important role in influenza virus neutralization, and the accumulation of vast numbers of viral particles in a few infected cells may facilitate lymphocytes such as natural killer (NK) cells to destroy the viruses and virus-infected cells (Fig. S6).

Previous studies have shown that therapeutic antibodies are effective in the treatment of various infectious diseases, such as SARS and influenza (12, 42). Oseltamivir, zanamivir, and amantadine are currently available therapeutics specifically for the treatment of influenza virus infections (45). However, mutations at the active sites of the drug-targeting proteins lead to reduced effects and resistant strains (44). Therefore, extensive efforts have been made to screen for new therapeutics, and some lead drugs with promising application prospects have been identified (17, 18, 45, 57). To assess the therapeutic potential of our nAbs, we prepared two humanized nAbs (C2D7 and CC4H4) and tested their therapeutic effects in mice infected with H7N9 virus. Excitingly, these two nAbs showed therapeutic efficacy, consistent with their neutralization capacity. C4H4, in particular, yielded a 100% survival rate even when the treatment was delayed until day 3 p.i., suggesting strong potential as an H7N9 therapeutic.

In conclusion, the nAbs 1H9, 2D7, and C4H4 have different neutralization capacities in terms of inhibiting the attachment, membrane fusion, and viral egress and release of H7N9 virus. The epitope of C4H4 consists of 24 amino acid residues and has extensive hydrogenic interactions with residues of the Fab, explaining its broad and strong neutralizing capacity. The nAbs 1H9 and 2D7 engage similar epitopes and cannot neutralize the HA G144E mutation-bearing SD001 strain. The humanized nAbs C2D7 and CC4H4 can completely protect mice infected with the deadly H7N9 virus, and CC4H4 is more effective if treatment is delayed. These findings provide a molecular basis for the rational design of vaccines and therapeutics against H7N9 viruses.

## MATERIALS AND METHODS

### Cells and viruses

Human lung carcinoma cells (A549) and Madin-Darby canine kidney cells (MDCK) were cultured in F-12K and DMEM mediums (Life Technologies Inc., Grand Island, NY, USA), respectively, supplemented with 10% fetal bovine serum (FBS), at 37°C in 5% CO_2_. Spodoptera frugiperda 21 cells (Sf21) and high five cells (Hi5) were suspension-cultured in Sf-900^TM^ III serum-free medium and Express Five serum-free medium (Gibco, Grand Island, NY, USA), respectively, at 28°C in shaker incubators.

Wild-type influenza viruses A/pigeon/Shanghai/S1069/2013 (H7N9) (S1069), A/duck/Guangxi/SDY129/2014 (H7N9) (SDY129), A/chicken/Guangdong/SD008/2017 (H7N9) (SD008), A/chicken/Yunnan/SD193/2017 (H7N9) (SD193), A/duck/Fujian/SD001/2018 (H7N9) (SD001), A/duck/Fujian/S4170/2014 (H7N9) (S4170), A/chicken/Shanghai/S1053/2013 (H7N9) (S1053), and A/duck/Zhejiang/S4488/2014 (H7N9) (S4488) were isolated and maintained by our laboratory.

### Amino acid sequence analysis of H7N9 HA

A total of 1, 305 full-length and nonredundant HA amino acid sequences of H7N9 influenza viruses were downloaded from the NCBI FLU database. Multiple sequence alignment was conducted with Geneious alignment in the Geneious Prime software (version 2022.2.2). Entropy analysis of HA sequences reveals the variation frequency at each amino acid site. A higher variation frequency correlates with an increased entropy value at the site. We filtered the amino acid sites with entropy values greater than 0.5 and used them to generate new sequences. We constructed a phylogenetic tree using these sequences and selected five H7N9 representative strains from different branches.

Furthermore, we also constructed the phylogenetic tree of all 1,305 full-length and nonredundant HA amino acid sequences. The trees in our study were constructed by using the neighbor-joining approach implemented in MEGA 6.0.6 with the default parameters. The tree topologies were evaluated by using 1,000 bootstrap analyses. We also indicated the locations of the five representative H7N9 viruses in the tree.

### Mouse immunization and monoclonal antibody (mAb) production

We chose the inactivated S4170, S1053, and S4488 strains as the immunogens to immune mice for generating mAbs. These strains were isolated by our laboratory from Fujian, Shanghai, and Zhejiang, China in 2014, 2013, and 2014, respectively. They were clustered into different genotypes (24).

The vaccination protocol is as follows: these viruses were propagated and titrated in the allantoic cavities of 10-day-old specific-pathogen-free (SPF) chicken embryonated eggs at 37°C for 48 h. One day before each immunization, the viruses were inactivated by using β-propiolactone at 0.1% (vol/vol) for 72 h at 4 °C and then emulsified in Freund’s incomplete adjuvant. The virus inactivation was confirmed by performing three serial passages of inactivated viruses in embryonated chicken eggs and then detecting it by hemagglutination test. The viruses were then concentrated using 100 kDa ultrafiltration tubes. We used 0.5% PBS-suspended chicken RBCs to test their hemagglutination titers and adjusted them to 8 Log2 hemagglutination titers using PBS.

Six-week-old female BALB/c mice were immunized by intraperitoneal injection. The mice received 500 µL of inactivated S4170, S1053, or S4488 viruses in sequence at 14-day intervals. Finally, 500 µL of inactivated S1053 virus was used as a booster immunization three days before fusion.

For the fusion step, mice were euthanized after booster immunization, and their spleens were collected in DMEM. Splenocytes were released by gently pressing the spleens between two frosted glass slides. Tissue debris was removed by gravity sedimentation, serially transferring splenocytes to fresh 15 mL centrifuge tubes. The splenocytes were mixed with myeloma Sp2/0 cells in a 50 mL centrifuge tube, and the cell culture supernatants were discarded after centrifugation. To induce cell fusion, 3 mL of polyethylene glycol was added to the cells and incubated for 3 min. The fusion reaction was terminated by adding 40 mL of DMEM. After centrifugation, the culture supernatants in cell mixtures were discarded. The hybridoma cells were distributed into 96-well plates pre-seeded with feeder cells. They were cultured in DMEM supplemented with 20% fetal bovine serum and 1% hypoxanthine-aminopterin-thymidine (HAT), at 37°C in 5% CO_2_.

The hybridoma cells were screened for the secretion of HA-specific mAbs by using HI and ELISAs. The nAbs were further screened by using an MN assay in MDCK cells. Three nAbs, 1H9, 2D7, and C4H4, neutralized all tested H7N9 viruses. Their hybridoma cells were cloned at least three times via limiting dilution, and the positive cells were expanded in culture. The nAbs were produced by injecting the hybridoma cells into BALB/c mice and collecting the ascites. The nAbs were purified from mouse ascites by using protein G agarose columns (GE Healthcare).

### Sequencing of the variable gene regions of the nAbs

Sequencing of the nAbs’ variable gene regions was performed as follows. Total RNA was extracted from 10⁷ hybridoma cells using TRIZOL reagent and reverse-transcribed using a reverse transcription kit (Transgen AE301-02, Beijing, China) with these primers: for the light chain variable region gene: 5′-CCGTTTGKATYTCCAGCTTGGTSCC-3′; for the heavy chain variable region gene: 5′-CGGTGACCGWGGTBCCTTGRCCCCA-3′. The cDNA of the variable gene regions was then amplified by PCR using a primer library from a previous study (58). Finally, the PCR products were sequenced on an Applied Biosystems 3500xL Genetic Analyzer (United States of America).

### Microneutralization (MN) assay

The MN assay was performed as previously described (59). Briefly, nAbs were serially 2-fold diluted from 100 µg/mL in Opti-MEM containing 2 µg/mL TPCK-trypsin (Sigma-Aldrich), mixed with an equal volume of 100 TCID_50_ (50% tissue culture infective doses) of the virus, and incubated in 96-well plates for 2 h at 37°C. After incubation, 4 × 10^4^ cells/well of MDCK cells were added to the 96-well plates. After incubation in an incubator supplied with 5% CO_2_ at 37°C for 48 h, 25 µL of culture supernatant was collected from each well, mixed with 75 µL of MU-NANA (Sigma-Aldrich), and incubated at 37°C for 1 h. The reaction was stopped by the addition of 100 µL of stop solution. Fluorescence was read on a Victor V plate reader (Perkin Elmer) with the following settings: excitation 355 nm, emission 460 nm, and 10 flashes per well. The half maximal inhibitory concentration (IC_50_) of each nAb is presented as the concentration that reduced the fluorescence signal by 50% compared with that of the control and calculated with the nonlinear fit algorithm curve fit in Graph Pad Prism 7.0.

### EC_50_ ELISA

Purified viruses (5 µg/mL) were immobilized in ELISA plates (50 µL per well) in sodium bicarbonate buffer (pH = 9.3) overnight. The reactions were blocked with PBS containing 5% wt/vol nonfat dry milk for 1 h at 37°C, and then, the viruses were captured by serial 2-fold dilutions of 1H9, 2D7, C4H4, or a control mAb, respectively. Plates were rinsed and incubated with horseradish peroxidase-conjugated goat-anti mouse IgG (H + L) (1: 5,000 dilution) (Sigma-Aldrich) at 25°C for 1 h. After the plates were washed, 100 µL of tetramethylbenzidine substrate was added to each well, and the plates were then incubated in a lightproof box at room temperature for 15 min, and the reaction was stopped with a 2 M H_2_SO_4_ solution. The absorbance was measured at 450 nm. All samples were tested in triplicate. The relative binding affinity to the purified viruses was measured as the concentrations for the half maximal effective concentration (EC_50_) of the nAbs.

### H7N9-infected cell binding assay

MDCK cells were inoculated with 1 MOI of SD008 in Opti-MEM I for 12 h. Then, the virus-infected cells were trypsinized and stained with 1H9, 2D7, C4H4, a control mAb, or a chicken-derived positive serum for 30 min at 4℃, and then with a secondary [Alexa Fluor 488 highly cross-adsorbed donkey anti-mouse IgG or a goat anti-chicken IgY (H + L), dilution 1:2,000] antibody (Thermo Fisher Scientific, Hillsboro, OR, USA) for 30 min at 4℃. The 4% paraformaldehyde (PFA) fixed cell suspensions were subjected to flow cytometry on a Cytomics TM FC500 flow cytometer (Beckman Coulter, USA). The data were analyzed using Flow Jo software (Flow Jo LLC).

### HI assay

The nAbs were 2-fold serially diluted in PBS in a 96-well V-bottom plate, mixed with 4 HA units of virus per well at room temperature for 30 min, and then 0.5% PBS-suspended chicken RBCs were added to each well. The highest dilution that prevented the agglutination of the RBCs was scored as the HI titer of the serum or nAb.

### Attachment inhibition assay

To assess whether the nAbs could inhibit the attachment of influenza virus, 1H9, 2D7, C4H4, and a control mAb at concentrations of 0.1, 1, 10, or 100 µg/mL, respectively, were mixed with 10 MOI of SD008 and incubated at 37°C for 1 h. The mixtures were added to A549 cells in six-well plates and inoculated on ice for 1 h. The A549 cells were then washed with pre-cooled PBS, and the attached viruses were detected by using flow cytometry, qPCR, and microscopy.

For flow cytometry, the cells were trypsinized, fixed with 4% PFA on ice for 30 min, permeabilized with 0.1% Triton X-100 at room temperature for 15 min, and then stained with a rabbit anti-NP mAb (Sino Biological, Beijing, China) and a secondary mAb Alexa Fluor 488 highly cross-adsorbed donkey anti-rabbit IgG (H + L) (Thermo Fisher Scientific, Hillsboro, OR, USA). The cell suspensions were analyzed by a Cytomics TM FC 500 flow cytometer (Beckman Coulter, USA). The data were analyzed using Flow Jo software (Flow Jo LLC).

For qPCR detection, the total RNA of H7N9-infected cells was isolated by using TRIZOL reagent and was reverse-transcribed by using a reverse transcription kit (Transgen AE301-02, Beijing, China) according to the manufacturer’s instructions. Relative mRNA expression levels were analyzed by using SYBR green qPCR Master Mix (Vazyme, Nanjing, China) with the following H7N9 NP gene-specific primers: SD008/NP FOR: 5′-CAGTGGCTCATAAATCCTG-3′ and SD008/NP REV: 5’- TGAGACTAAAGACCTGGCTGT-3′. The 2^−ΔΔCt^ method was used to calculate the relative gene expression level. The 28S rRNA (A549 cells) gene was amplified as an internal control by using the primers: 28S rRNA FOR: 5′-CAGACATTTTGCTCTCAAGCTG-3′ and 28S rRNA REV: 5′-GGGTGGTAAACTCCATCTAAGG-3′.

For confocal microscopy, A549 cells were cultured on Millicell EZ SLIDE 4-well Glass slides (Merck Millipore, Darmstadt, Germany). After viral attachment, the cells were immediately fixed with 4% PFA for 15 min, permeabilized with 0.1% Triton X-100 at room temperature for 10 min, blocked with 5% wt/vol non-fat dry milk for 30 min, and stained with the corresponding primary mAb (rabbit anti-NP mAb) (Sino Biological, Beijing, China) overnight at 4°C and the secondary mAb [Alexa Fluor 488 highly cross-adsorbed donkey anti-rabbit IgG (H + L)] (Thermo Fisher Scientific, Hillsboro, OR, USA) at room temperature for 1 h. Nuclei were stained with 4’,6-diamidino-2-phenylindole (DAPI). Fluorescence intensity was quantified with a Zeiss LSM880 laser-scanning confocal microscope (Carl Zeiss Meditec, Jena, Germany). The resolution of the acquired images was 1,024 × 1,024. The cell-bound virus signal intensities were quantified from at least 110 cells for each sample by ZEN software.

### Syncytium inhibition assay

To assess whether the nAbs could inhibit the membrane fusion of influenza virus, A549 cells seeded in six-well plates were infected with 1 MOI of SD008, treated with 2 µg/mL TPCK-trypsin for 15 min at 24 h, and inoculated with Opti-MEM I containing 1H9, 2D7, C4H4, or a control mAb at gradient concentrations of 0.1, 1, 10, or 100 µg/mL at 37℃ for 30 min. The cells were treated with sodium citrate buffer (pH = 5.0) at 37℃ for 2 min. Then, the cell cultures were removed, and the cells were neutralized with a complete growth medium at 37℃ for 3 h, fixed with 4% PFA for 30 min, and stained with the rabbit anti-HA mAb (Invitrogen) and Alexa Fluor 488 highly cross-adsorbed donkey anti-rabbit IgG (H + L) mAb (Thermo Fisher Scientific, Hillsboro, OR, USA). Cell nuclei were stained with DAPI. The cells were visualized and photographed by using an inverted fluorescence microscope (EVOS M5000 Cell Imaging System, Life Technologies).

### HA cleavage inhibition assay

We analyzed the inhibitory ability of nAbs on the cleavage of recombinant HA protein by TPCK-trypsin. TPCK-trypsin cleaves the recombinant HA protein into HA1 and HA2, which remain covalently linked by a disulfide bond. We assessed the degree of cleavage by detecting the remaining intact recombinant HA protein in the sample using SDS-PAGE. SDS destroys the disulfide bond between HA1 and HA2, separating them. Gradually weakening HA bands over time indicate negative results, meaning the nAbs have no inhibitory ability. Conversely, unchanged HA bands over time indicate positive results, showing that the nAbs have inhibitory ability.

To select a suitable TPCK-trypsin concentration for the HA cleavage inhibition assay, 10 µg of the recombinant SD008 HA protein was treated with TPCK-trypsin at the concentrations of 2.5, 5, or 10 µg/mL at 37℃ for 0, 5, 10, 20, or 40 min, respectively. Reactions were stopped by the addition of a 5× SDS loading buffer. The protein samples were analyzed by using SDS-PAGE.

Inhibition of HA cleavage by Trypsin cleavage inhibition assay was performed by mixing 10 µg of the recombinant HA protein with 1H9, 2D7, C4H4, or a control mAb at a molar ratio of 1.5:1 (mAb:HA monomer) and incubated at 37℃ for 1 h. TPCK-trypsin at the concentration determined in the above assay was mixed with the antibody-HA mixtures, and incubated for 0, 5, 10, 20, or 40 min at 37℃. Reactions were stopped by the addition of 5× SDS loading buffer. The protein samples were boiled for 10 min and analyzed using SDS-PAGE.

### HA conformational change inhibition assay

To assess the potential of the nAbs to inhibit the HA conformational change induced by a low pH environment, 10 µg of SD008 HA protein was incubated with 1H9, 2D7, C4H4, or a control mAb at a molar ratio of 1.5:1 (mAb:HA monomer) at 37℃ for 1 h to form HA-nAb complexes, and then, 1% dodecylmaltoside was added prevent protein aggregation. The reaction solution was acidized with 100 mM sodium acetate buffer (pH = 5.0) at 37°C for 1 h and neutralized with tris buffer (pH = 8.0). TPCK-trypsin was added at an HA:TPCK-trypsin ratio of 20:1 (WT:WT) and incubated at 37°C for 30 min. The reactions were stopped by the addition of 5 × nonreducing SDS loading buffer and protein samples were analyzed by using SDS-PAGE.

### RBC lysis inhibition assay

Under TPCK-trypsin and low pH conditions, the conformationally changed HA protein with exposed fusion peptide leads to the disruption of RBC membranes, resulting in the leakage of RBC contents, including nicotinamide adenine dinucleotide phosphate (NADPH). By detecting NADPH, we can assess the degree of RBC leakage and the inhibitory effect of the nAbs on this process.

For the RBC lysis inhibition assay, 1 × 10^7^ TCID_50_ of SD008 virus was incubated with SPF chicken RBCs (2% final concentration) on ice for 10 min. The nAbs were serially 2-fold diluted from 100 µg/mL, mixed with an equal volume of the virus-RBC mixtures, and incubated on ice for 30 min. Sodium citrate buffer (pH = 4.6) was added to the samples to reach a final pH of 5.0, and the samples were then incubated at room temperature for 30 min. The samples were centrifuged at 2,000 *g* for 10 min. Then, 100 µL of the supernatant was added to an ELISA plate to determine the NADPH content by optical density measurement (340 nm).

### Egress inhibition assay

The assay was carried out as described previously (60). In brief, monolayers of MDCK cells (2 × 10^5^ cells/well) were seeded in 12-well plates and cultured in DMEM supplied with 10% FBS overnight at 37℃. The cells were then washed with PBS and infected with 1 MOI of SD008 for 2 h at 37℃. The cells were rinsed six times with PBS to remove the noninternalized viruses. The nAbs, at an initial concentration of 100 µg/mL, or oseltamivir at 1 µM were 10-fold serially diluted and added to the cells together with 25 mM NH_4_Cl to prevent *de novo* infection (61). Cell supernatants were harvested at 24 h p.i. to quantify the NP gene by using RT-qPCR with the H7N9 NP primers and a probe (SD008/NP probe: 5′-FAM-CTACCTATCAGAGAACGAGA-BHQ1-3’) and the NA protein by using the method described in the MN assay.

### Transmission electron microscopy (TEM)

To examine nAb inhibition of H7N9 viral egress, we infected MDCK cells with 1 MOI SD008 for 2 h at 37℃, discarded the cell culture medium, and rinsed the cells six times with PBS to remove noninternalized viral particles. Fresh Opti-MEM I cultures containing nAbs (100 µg/mL) or oseltamivir (1 µM) were added and incubated for another 22 h. The cells were washed three times with PBS, scraped off the plate, and pelleted at 13,000 *g* for 15 min. The pellets were used to prepare ultra-thin sections following the standard procedure. The EM samples were examined on a transmission electron microscope (H-7650, Hitachi, Tokyo, Japan), operated at 80 kV.

To locate the nAbs in the cells, pre-embedding immunogold EM samples were prepared. Briefly, MDCK cells were infected and treated with the nAbs for 21 h. The cell culture medium was discarded, and the cells were rinsed three times with PBS. Fresh Opti-MEM I cultures containing goat anti-mouse IgG conjugated with 10 nm gold particles (EMS, 25208) were added and incubated for another 1 h. After being washed three times with PBS, the cells were scraped off and pelleted to prepare ultrathin sections.

### Recombinant HA ectodomain expression and production

In brief, the gene of the SD008 HA ectodomain was synthesized with a gp67 signal peptide gene linked to its N-terminal end, a thrombin cleavage site sequence, a trimeric foldon sequence, and a 6 × His tag sequentially linked to the C-terminal end. HA proteins were produced by the Bac-to-Bac Baculovirus Expression System (Invitrogen). Baculovirus was rescued in Sf21 cells. The soluble SD008 HA protein was expressed in Hi5 cells and purified through a HisTrap HP 5 mL column (GE Healthcare, USA). The protein was treated with thrombin (Sigma-Aldrich) and further purified by use of a Superdex 200 increase 10/300 Gl column (GE Healthcare, USA).

### Preparation of HA-Fab complexes

To prepare the Fabs, the nAbs were cleaved by using the Pierce Fab preparation kit (Thermo Fisher Scientific, Hillsboro, OR, USA) according to the manufacturer’s instructions. Briefly, 4 mg of each nAb in a volume of 0.5 mL was cleaved with papain for 5 h at 37℃. The Fabs were purified and isolated by inoculating the samples with a NAb Protein A Plus Spin Column at room temperature for 10 min. The HA-Fab complexes were prepared by incubating the two proteins at a monomer HA-to-Fab molar ratio of 1:1.5 at 4℃ for 1 h and purified by using a Superdex 200 increase 10/300 Gl column.

### Cryo-EM sample preparation and data acquisition

For each sample, 3.5 µL of the purified protein sample (0.4 mg/mL) was applied onto an O_2_/Ar glow-discharged 200 mesh Quantifoil R1.2/1.3 grid (Quantifoil, Micro Tools GmbH, Germany). The grid was blotted for 5s with a blotting force of −4 and vitrified by plunge-freezing into liquid ethane by using a Vitrobot Mark IV (Thermo Fisher Scientific, Hillsboro, OR, USA) at 4°C under 100% humidity. Cryo-EM data were collected on a 300 keV Titan Krios electron microscope (Thermo Fisher Scientific, Hillsboro, OR, USA) equipped with a direct electron detector GIF-K2 Summit camera (Gatan, USA). Images were recorded at 1,30,000× magnification and calibrated at a super-resolution pixel size of 1.076 Å/pixel. The exposure time for the HA trimer was set to 4.88 s with a total accumulated dose of 50 electrons per Å^2^. The samples of complexes were collected at 0° and 40° tilts with an exposure time of 8.38 s and a total accumulated dose of 60 electrons per Å^2^. All images were automatically recorded by using SerialEM and were collected with a defocus range from −3 μm to −1.5 µm. Statistics for data collection and refinement are provided in Table S1. The methods for processing are described in Fig. S4.

### Cryo-EM data processing and 3D reconstruction

A total of 623, 1,620, 1,636, and 1,941 movie stacks were collected for HA, HA-1H9 Fab, HA-2D7 Fab, and HA-C4H4 Fab, respectively. After beam-induced drift correction, contrast transfer function (CTF) parameter estimation, particle auto-picking, and manual particle checking, reference-free 2D classification was performed for single particle analysis in cryoSPARC V3 (62). A total of 93,530 particles in the HA data set remained after 2D classification, and then, 61,557 particles with better structural features were selected for 3D auto-refinement. Finally, the EM map of the HA trimer was obtained and resolved to a resolution of 3.1 Å with C3 symmetry imposed. To overcome the preferred orientation of the complexes, the micrographs collected at 0° and 40° tilts were combined for the subsequent analyses. After 2D and 3D classifications, a total of 179,237, 208,155, and 150,008 particles corresponding, respectively, to the HA-1H9 Fab, HA-2D7 Fab, and HA-C4H4 Fab complexes remained and were further screened according to the saturability of the complexes with symmetry expansion. The remaining 125,242, 160,967, and 112,251 particles corresponding, respectively, to the HA-1H9 Fab, HA-2D7 Fab, and HA-C4H4 Fab complexes were used to perform homogeneous refinement, local refinement, and global CTF refinement. The final EM maps of the HA-1H9 Fab, HA-2D7 Fab, and HA-C4H4 Fab complexes were resolved to resolutions of 2.9 Å, 3.0 Å, and 2.9 Å, respectively, with C3 symmetry imposed.

### Atomic model building, refinement, and 3D visualization

To build the atomic models of the HA trimer and the complexes, their homology models were generated by using the SWISS MODEL server (https://swissmodel.expasy.org) and fitted by rigid-body refinement into the EM maps by using UCSF Chimera (63). Then, the atomic model coordinates were further corrected and adjusted by real-space refinement in Coot (41). Finally, under minimization global, local grid search, and ADP conditions, the last round of flexible fitting of the entire complexes was performed in PHENIX (64). The refinement statistics of the structural models are listed in Table S1. All the cryo-EM figures and statistics related to the surface potential calculations were finished by using the APBS server (https://apbs.readthedocs.io/en/latest/) and generated with UCSF Chimera (63). The interaction surface analysis was performed on the web PISA server (https://www.ebi.ac.uk/msd-srv/prot_int/pistart.html).

### The 50% mouse lethal dose (MLD_50_)

Groups of mice (Vital River Laboratories, Beijing, China) (*n* = 5 per group) were inoculated intranasally with 10-fold serial dilutions containing 10^1^–10^6^ TCID_50_ of CK/SD008-PB2/627K H7N9 virus in a volume of 50 µL, respectively. The mice were monitored for 14 days for weight loss and mortality. The MLD_50_ of CK/SD008-PB2/627K H7N9 virus was calculated by using the method of Reed and Muench.

### Preparation of recombinant humanized nAbs

The humanized nAbs were prepared as previously described (12). The variable heavy- and light-chain regions of 2D7 and C4H4 were synthesized as Homo sapiens codon-optimized gene fragments. These optimized genes were then combined with human IgG gamma heavy chain (GenBank: FJ716123.1) or kappa light chain (GenBank: FJ475056.1) by PCR. Finally, each of the resulting recombinant genes was cloned into a pCDNA3.1 vector. To obtain these two chimeric-expressed humanized nAbs, the CHO-K cells were co-transfected by recombinant plasmids of heavy or light chains of nAbs. The humanized nAbs (named C2D7 and CC4H4) were purified from the supernatant of cell cultures by use of protein G affinity chromatography (GE Healthcare, USA).

### Treatment of H7N9-infected mice with humanized nAbs

To assess the therapeutic effect of C2D7 and CC4H4 on H7N9 infection in mice, we designed treatment strategies to assess the effect of normal (treated on day 1 p.i.) and delayed (treated on day 3 or 5 p.i.) treatments. All the mice were infected with 25 MLD_50_ of an H7N9 virus (CK/SD008-PB2/627K), a lethal strain in mice.

In the normal treatment trial, a total of 86 female BALB/c mice aged 6 weeks were divided into seven groups: four groups (*n* = 11 for each) were intravenously administered 0.1 or 1 mg/kg C2D7 or CC4H4 at 24 h post-infection (p.i.), and three groups (*n* = 14 for each) were intravenously administered 10 mg/kg C2D7, CC4H4, or an unrelated control mAb at 24 h p.i.. The mortality and body weight of the mice were recorded daily for up to day 14 p.i.. Mice that lost more than 25% of their initial body weight were euthanized in accordance with our animal ethics protocol. Furthermore, three mice in each group were euthanized on days 3 and 6 p.i., respectively, and their lungs were collected and titrated for virus loading in MDCK cells. The lungs of three mice in each of the C2D7 (10 mg/kg), CC4H4 (10 mg/kg), and control mAb (10 mg/kg) groups were collected on day 6 p.i. to examine pathogenic damage by performing HE staining.

In the delayed treatment trials, 30 mice were divided into six groups and intravenously administered 10 mg/kg C2D7 or CC4H4, or orally administered 25 mg/kg oseltamivir on day 3 or 5 p.i.. The mice were observed daily for mortality and body weight up to day 14 p.i.. Mice that lost more than 25% of their initial body weight were euthanized in accordance with our animal ethics protocol.

### Quantification and statistical analysis

EC_50_ and IC_50_ values were determined after log transformation of concentration values and nonlinear regression analysis. Other experimental data were analyzed statistically by using a one-way ANOVA with Dunnett’s multiple comparisons test (ns: not significant; *P < 0.05; **P < 0.01; ***P < 0.001; ****P < 0.0001). Statistical analyses were performed with GraphPad Prism 7 software.

## Data Availability

Cryo-EM maps for the SD008 HA trimer, SD008 HA-1H9 Fab, SD008 HA-2D7 Fab, and SD008 HA-C4H4 Fab have been deposited at the Electron Microscopy Data Bank (EMDB) with the following accession codes: EMD-35729, EMD-35733, EMD-35734, and EMD-35735, respectively. The atomic coordinates have been deposited at the Protein Data Bank (PDB) with the following accession codes: 8IUX, 8IUY, 8IUZ, and 8IV0. Source data are provided in this paper.

## SUPPLEMENTAL MATERIAL

Fig. S1 jvi.01400-24-s0001.tif Annotation of the amino acid sequence of the nAbs and phylogeny of representative H7N9 viruses.

Fig. S2 jvi.01400-24-s0002.tif The nAbs inhibit H7N9-induced syncytium formation in A549 cells.

Fig. S3 jvi.01400-24-s0003.tif Transmission electron microscopy (TEM) observations elaborate on the egress inhibition mechanism of the nAbs.

Fig. S4 jvi.01400-24-s0004.tif Workflow of cryo-electron microscopy (cryo-EM) image processing of the SD008 HA trimer, and the complexes of HA-1H9 Fab, HA-2D7 Fab, and HA-C4H4 Fab.

Fig. S5 jvi.01400-24-s0005.tif Structural superimposition and root mean square deviation (RMSD) calculation between SD008 HA and other H7 HAs.

Fig. S6 jvi.01400-24-s0006.tif Schematic diagram showing the neutralizing mechanisms of the three nAbs.

Supplemental material jvi.01400-24-s0007.pdf Tables S1 to S7; legends for Fig. S1 to S6.

ASM does not own the copyrights to Supplemental Material that may be linked to, or accessed through, an article. The authors have granted ASM a non-exclusive, world-wide license to publish the Supplemental Material files. Please contact the corresponding author directly for reuse.
