# Supplementary figures and images for: Structural basis of different neutralization capabilities of monoclonal antibodies against H7N9 virus
Source: J Virol. 2024 Dec 20;99(1):e01400-24. doi: 10.1128/jvi.01400-24 (PMC11784312; doi:10.1128/jvi.01400-24)

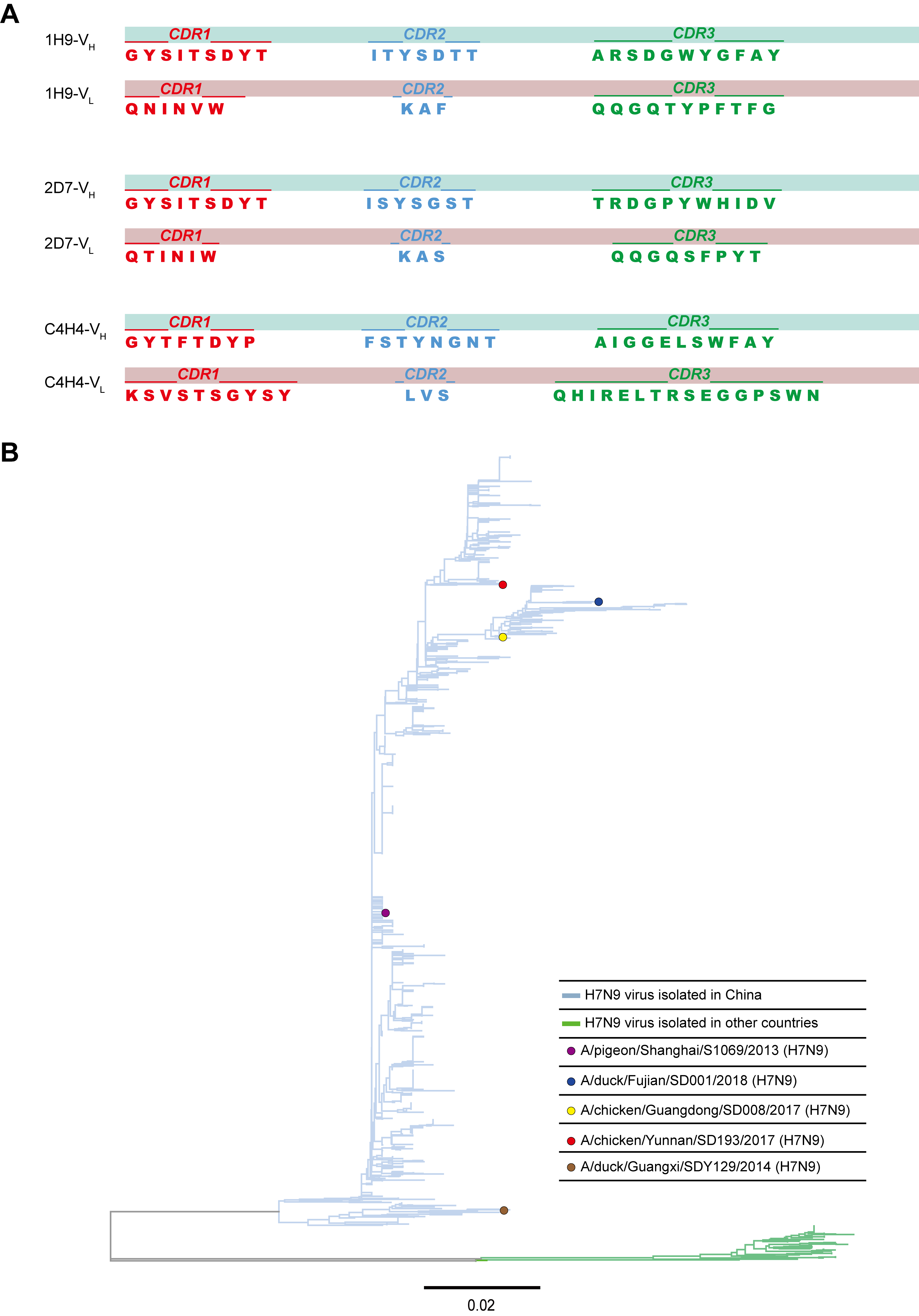

Supplement: Fig. S1 — Annotation of the amino acid sequence of the nAbs and phylogeny of representative H7N9 viruses. [file jvi.01400-24-s0001.tif]

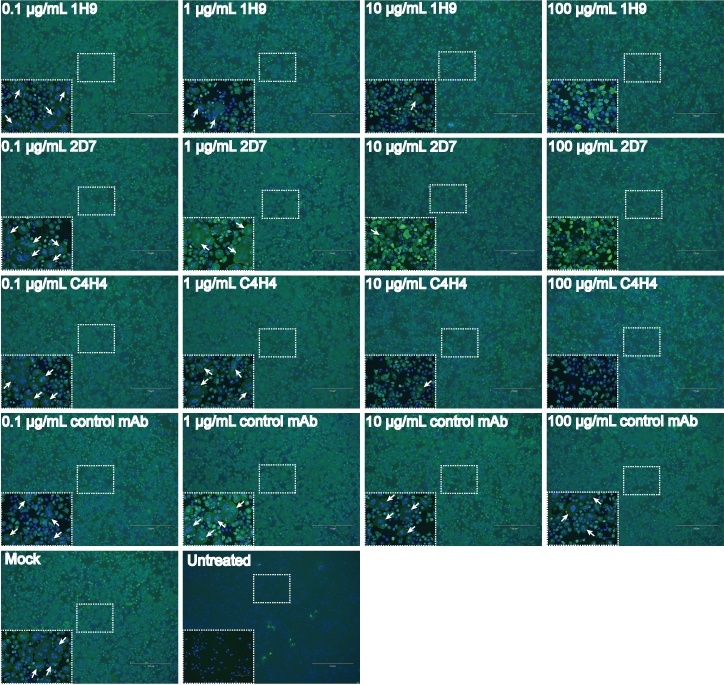

Supplement: Fig. S2 — The nAbs inhibit H7N9-induced syncytium formation in A549 cells. [file jvi.01400-24-s0002.tif]

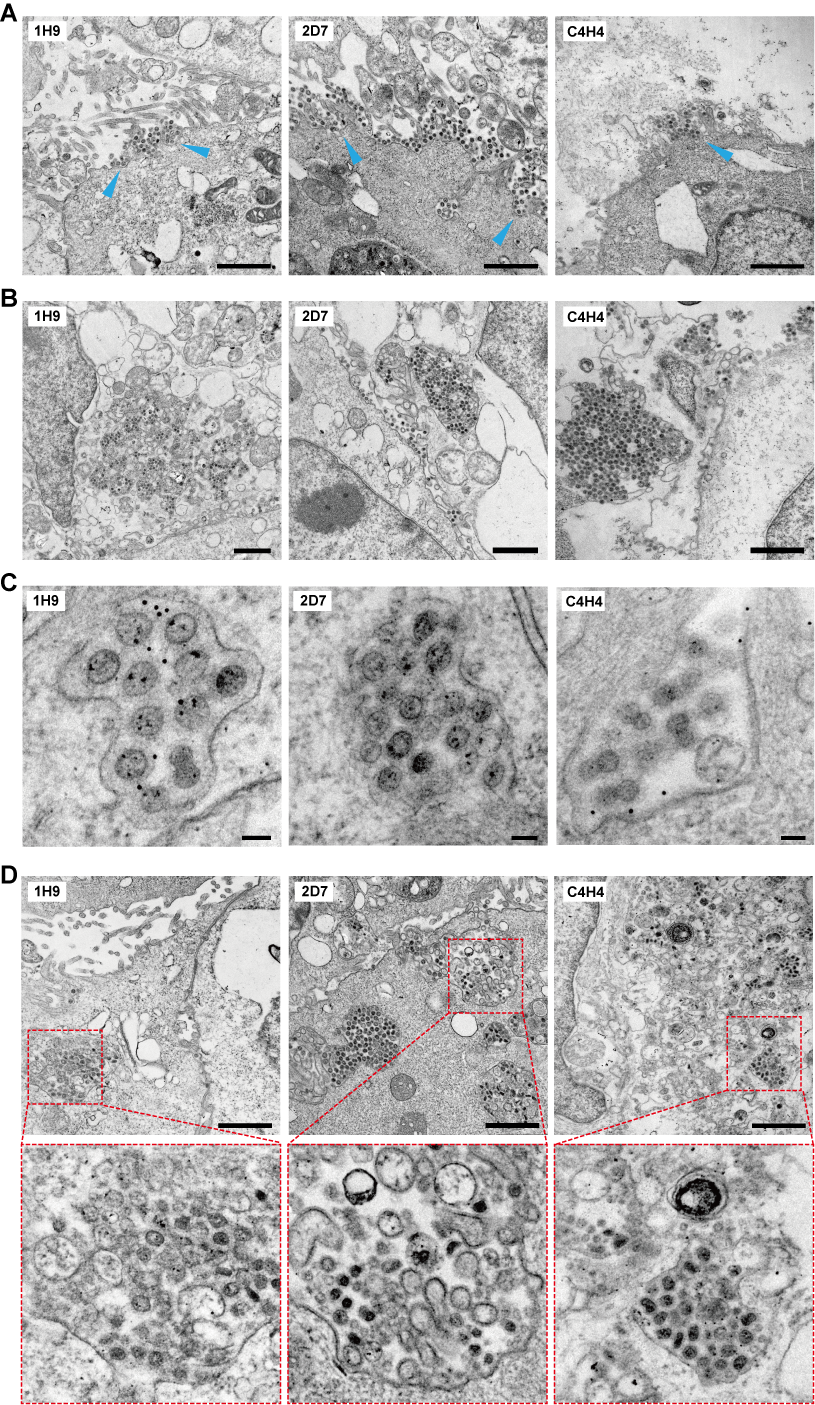

Supplement: Fig. S3 — Transmission electron microscopy (TEM) observations elaborate on the egress inhibition mechanism of the nAbs. [file jvi.01400-24-s0003.tif]

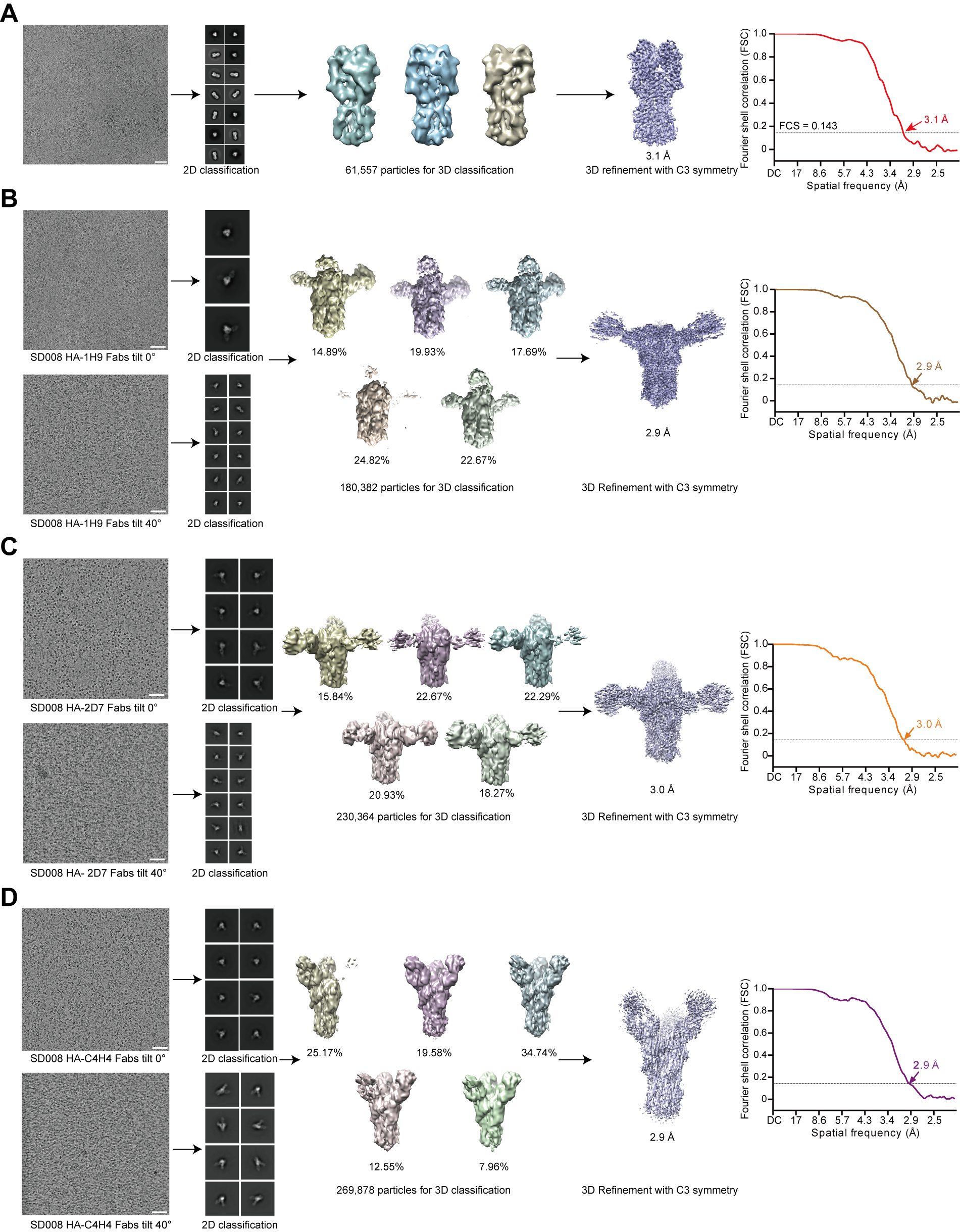

Supplement: Fig. S4 — Workflow of cryo-electron microscopy (cryo-EM) image processing of the SD008 HA trimer, and the complexes of HA-1H9 Fab, HA-2D7 Fab, and HA-C4H4 Fab. [file jvi.01400-24-s0004.tif]

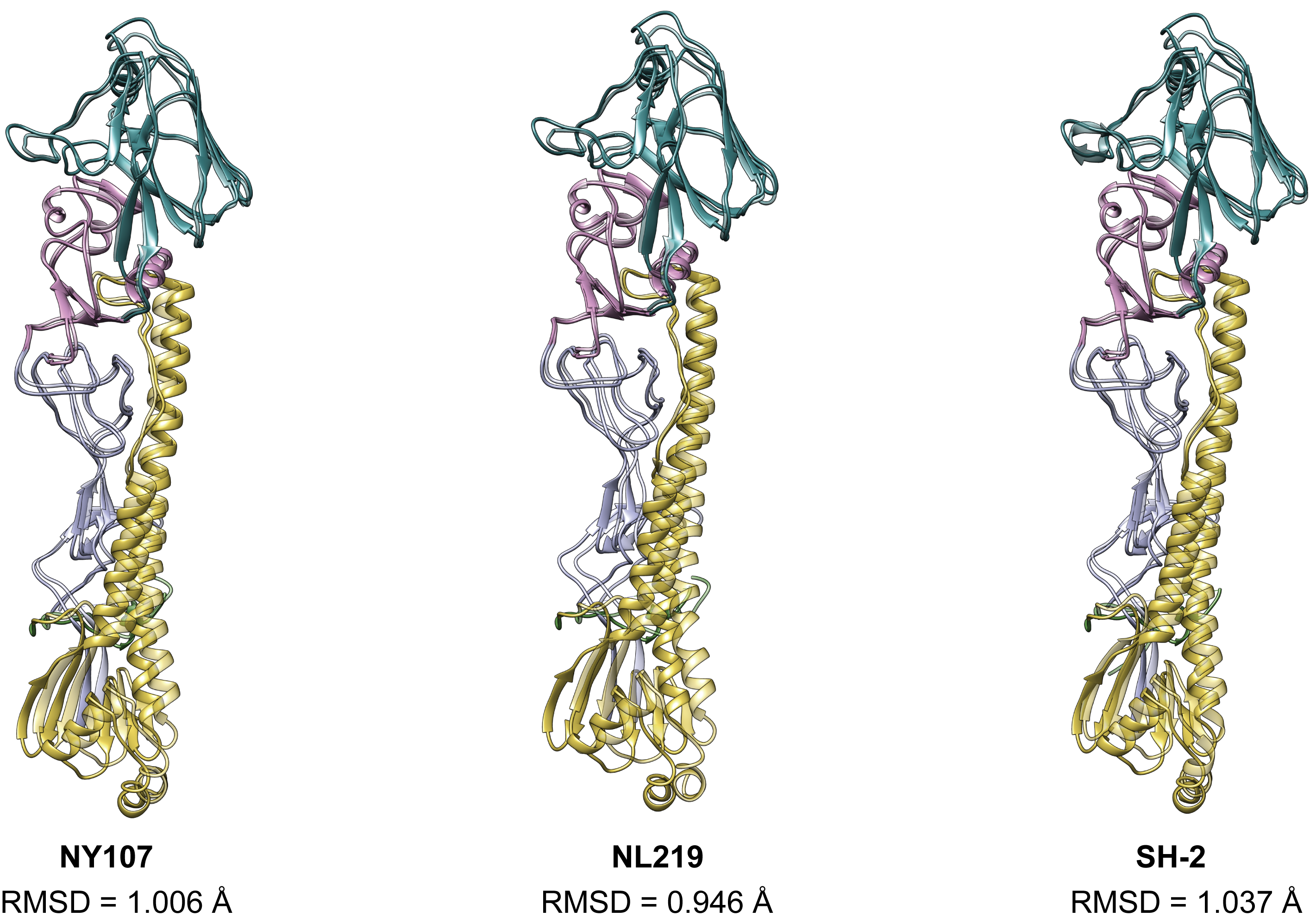

Supplement: Fig. S5 — Structural superimposition and root mean square deviation (RMSD) calculation between SD008 HA and other H7 HAs. [file jvi.01400-24-s0005.tif]

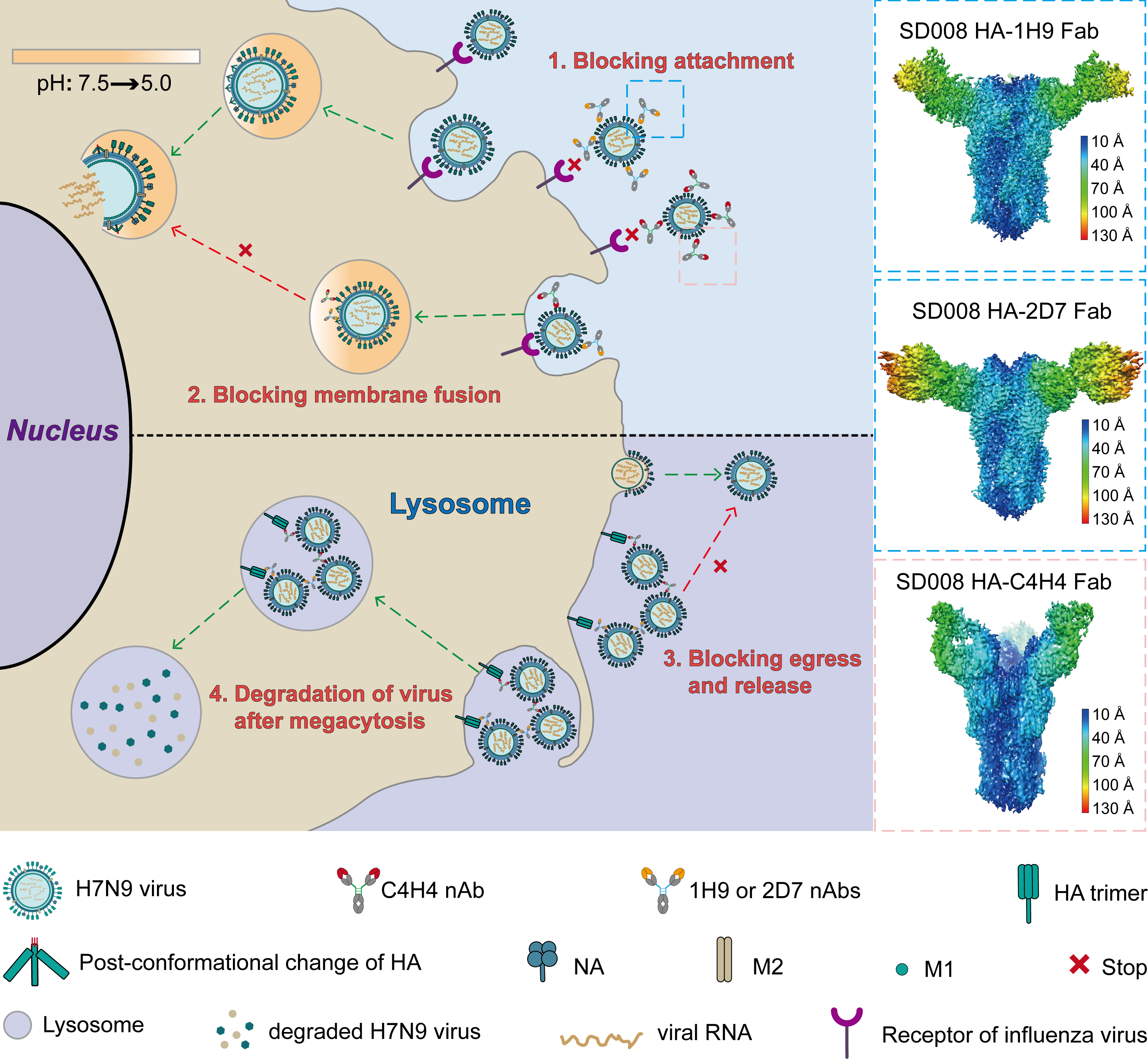

Supplement: Fig. S6 — Schematic diagram showing the neutralizing mechanisms of the three nAbs. [file jvi.01400-24-s0006.tif]
