# Supplementary material for: Structural basis of different neutralization capabilities of monoclonal antibodies against H7N9 virus
Source: J Virol. 2024 Dec 20;99(1):e01400-24. doi: 10.1128/jvi.01400-24 (PMC11784312; doi:10.1128/jvi.01400-24)
Supplement: Supplemental material — Tables S1 to S7; legends for Fig. S1 to S6. [file jvi.01400-24-s0007.pdf]

SUPPLEMENTAL FIGURES LEGENDS AND TABLES

Structural Basis of Different Neutralization Capabilities of Monoclonal Antibodies against H7N9 Virus

Bingbing Zhao,[a] Zhenzhao Sun,[a] Shida Wang,[a] Zhibin Shi,[a] Yongping Jiang,[a] Xiurong Wang,[a] Guohua Deng,[a] Peirong Jiao,[b] Hualan Chen,[a] Jingfei Wang[a*]

[a]State Key Laboratory for Animal Disease Control and Prevention & National Data Center for Animal Infectious Diseases, Harbin Veterinary Research Institute, Chinese Academy of Agricultural Sciences, Harbin 150069, People's Republic of China.

[b]College of Veterinary Medicine, South China Agricultural University, Guangzhou, 510642, People's Republic of China.

[*]Correspondence to: Jingfei Wang, wangjingfei@caas.cn

SUPPLEMENTAL FIGURES LEGENDS

FIG S1 Annotation of the amino acid sequence of the nAbs and phylogeny of representative H7N9 viruses. (A) The amino acid sequences of the heavy chain variable region (V_H) and light chain variable region (V_L) of 1H9, 2D7, and C4H4 were analyzed by using the IMGT database (http://www.imgt.org/). Complementarity-determining regions (CDR1, CDR2, and CDR3) are indicated in red, blue, and green, respectively. (B) The phylogenetic tree shows the phylogenetic relationship of the HA sequence of 1,305 H7N9 viruses. The tree was generated with the neighbor-joining method implemented in MEGA 6.0.6 and evaluated by 1,000 bootstrap analyses. The colored dots indicate the representative strains used in this study.

FIG S2 The nAbs inhibit H7N9-induced syncytium formation in A549 cells. A549 cells were infected with 1 MOI of SD008 for 24 h, treated with 1H9, 2D7, C4H4, or an unrelated mAb at different concentrations at 37°C for 30 min, cultured at a low pH (pH = 5.0) for 2 min, and then in normal cultures at 37°C for another 3 h. The cells were stained with a rabbit anti-HA mAb (Invitrogen) and the Alexa Fluor 488 highly cross-adsorbed donkey anti-rabbit IgG (H+L) mAb (Thermo Fisher Scientific, Hillsboro, OR, USA). Cell nuclei were stained with DAPI. White arrows point to syncytia.

FIG S3 Transmission electron microscopy (TEM) analysis of nAb-treated cells during the viral budding process. (A) Numerous viral particles are retained on the surface of the infected cell and are likely to be invaginated by cellular processes, as indicated by the blue triangles. Scale bar represents 1 μm. (B) Endocytic vesicles contain numerous viral particles. Scale bar represents 1 μm. (C) Pre-embedding immunogold labeling demonstrates that viral particles are endocytosed from the cell's exterior. Scale bar represents 100 nm. (D) Cytoplasmic vesicles contain numerous lysosomes. The insets show the enlarged view of the vesicles.

FIG S4 Workflow of cryo-electron microscopy (cryo-EM) image processing of the SD008 HA trimer (A), and the complexes of HA-1H9 Fab (B), HA-2D7 Fab (C), and HA-C4H4 Fab (D).

FIG S5 Structural superimposition and root mean square deviation (RMSD) calculation between SD008 HA and other H7 HAs. The NY107, NL219, and SH-2 HAs had been reported previously.

FIG S6 Schematic diagram showing the neutralizing mechanisms of the three nAbs. The scale bars show the distance from any point to the Z-axis of the HA trimer.

SUPPLEMENTAL TABLES

TABLE S1 Data collection and refinement statistics for SD008 HA trimer and HA-Fab complexes.

	HA trimer	HA-1H9 Fab	HA-2D7 Fab	HA-C4H4 Fab
**Data collection and reconstruction**				
EMDB code	EMD-35729	EMD-35733	EMD-35734	EMD-35735
Microscope	Tian krios	Tian krios	Tian krios	Tian krios
Voltage (kV)	300	300	300	300
Detector	K2	K2	K2	K2
Dose rate (e/pixel/s)	10.245	7.160	7.160	7.160
Exposure (s)	4.88	8.38	8.38	8.38
Dose (e/Å)	50	60	60	60
Frames	32	32	32	32
Defocus range (μm)	-1.5~-3	-1.5~-3	-1.5~-3	-1.5~-3
Particles	61,557	179,237	160,967	112,251
B factor	-95	-55.8	-57.3	-49.5
Resolution (Å)	3.1	2.9	3.0	2.9
Reconstruction	CyroSPARC	CyroSPARC	CyroSPARC	CyroSPARC
PDB code	8IUX	8IUY	8IUZ	8IV0
R.m.s.d, bonds (Å)	0.010	0.010	0.010	0.010
R.m.s.d, angles (°)	1.000	1.000	1.000	1.000
**Ramachandran statistics**				
Favored (%)	95.53	89.89	88.45	90.67
Allowed (%)	4.47	10.11	11.55	9.22
Outliers (%)	0.00	0.00	0.00	0.11
Rotamer outliers (%)	0.48	1.01	0.76	0.51
Clashcore	2.87	5.85	7.35	8.84

TABLE S2 Hydrogen bond and salt bridge interactions between SD008 HA and 1H9 Fab by PISA Program.

HA	1H9	Chains[a]	BSA($Å^2$)[b]	Type[c]	Dist.(Å)[d]
ARG140[NH1]	ASP33[OD2]	V_H	343.6	S	2.57
ARG140[NH1]	ASP33[OD2]	V_H		H	2.57
SER143[OG]	ASP108[O]	V_H		H	2.23
SER143[OG]	TRP110[N]	V_H		H	2.94
SER145[OG]	ASP33[OD2]	V_H		H	3.34
SER145[OG]	ASP33[OD1]	V_H		H	3.39
ASP77[O]	ASN30[ND2]	V_L	373.8	H	3.27
ASP77[O]	TRP32[NE1]	V_L		H	3.66

[a]V_H: heavy chain variable region; V_L: light chain variable region.

[b]BSA: Buried surface area.

[c]H: Hydrogen bond; S: Salt bridge.

[d]Dist.: distance between the interacting atoms.

TABLE S3 Amino acids involved in the contacts between SD008 HA and 1H9 Fab (PISA).

Residues of SD008 HA[a]	Domain[b]	Residues of 1H9 Fab	Chain[c]
V135	RBD	Y113	V_H
R140	RBD	D33, Y58	V_H
R141	RBD	W110	V_H
S143	RBD	T35, D108, G109, W110	V_H
G144	RBD	D33, Y34	V_H
S145	RBD	D33, Y34	V_H
S146	RBD	Y113	V_H
D77	VED	N30, W32, Q108	V_L
Q78	VED	N30, Q108	V_L
F79	VED	N30	V_L
L80	VED	V31	V_L
R141	RBD	W32	V_L
Y148	RBD	K56	V_L
R256	RBD	V31, F58	V_L

[a]All the amino acids are shown according to H3N2 HA numbering.

[b]RBD: receptor binding domain; VED: vestigial esterase domain.

[c]V_H: heavy chain variable region; V_L: light chain variable region.

TABLE S4 Hydrogen bond and salt bridge interactions between SD008 HA and 2D7 Fab by PISA Program.

**HA**	**2D7**	**Chains**[a]	**BSA(Å^2)**[b]	**Type**[c]	**Dist.(Å)**[d]
ARG140[NH1]	ASP33[OD2]	V_H	367.8	S	2.96
ARG140[NH1]	ASP33[OD2]	V_H		H	2.96
ARG140[NH2]	ASP33[OD2]	V_H		S	3.90
SER143[OG]	GLY108[O]	V_H		H	2.43
SER145[OG]	ASP33[OD2]	V_H		H	3.44
SER145[O]	TYR34[OH]	V_H		H	3.78
ASP77[O]	TRP32[NE1]	V_L	359.0	H	3.63
ASN133[OD1]	TYR55[OH]	V_L		H	3.75
ALA149[O]	LYS56[NZ]	V_L		H	3.42

[a]V_H: heavy chain variable region; V_L: light chain variable region.

[b]BSA: Buried surface area.

[c]H: Hydrogen bond; S: Salt bridge.

[d]Dist.: distance between the interacting atoms.

TABLE S5 Amino acids involved in the contacts between SD008 HA and 2D7 Fab (PISA).

Residues of SD008 HA[a]	Domain[b]	Residues of 2D7 Fab	Chain[c]
R140	RBD	D33, Y58	V_H
R141	RBD	Y110, W113	V_H
S143	RBD	D33, Y34, G108, P109	V_H
G144	RBD	D33, Y34	V_H
S145	RBD	D33, Y34	V_H
S146	RBD	P109	V_H
D77	VED	N30, W32	V_L
Q78	VED	Q106	V_L
L80	VED	I31	V_L
E81	VED	N30, I31, S83	V_L
N133	RBD	Y55	V_L
A149	RBD	K56	V_L
K152	RBD	K56, N66	V_L
R256	RBD	I31	V_L

[a]All the amino acids are shown according H3N2 HA numbering.

[b]RBD: receptor binding domain; VED: vestigial esterase domain.

[c]V_H: heavy chain variable region; V_L: light chain variable region.

TABLE S6 Hydrogen bond and salt bridge interactions between SD008 HA and C4H4 Fab by PISA Program.

HA	C4H4	Chains[a]	BSA(Å^2)[b]	Type[c]	Dist.(Å)[d]
ASN128[ND2]	GLU109[OE1]	V_H	770.1	H	3.89
ASP157[N]	GLN69[OE1]	V_H		H	3.60
ASN157[OD1]	ASN66[ND2]	V_H		H	3.42
ASN158[N]	GLN69[OE1]	V_H		H	2.78
ALA159[N]	GLN69[OE1]	V_H		H	2.85
ALA160[N]	ASN66[OD1]	V_H		H	2.88
ALA160[O]	ASN66[ND2]	V_H		H	2.78
GLN163[O]	THR58[OG1]	V_H		H	3.10
THR165[N]	TYR59[OH]	V_H		H	2.69
THR165[N]	ASN60[OD1]	V_H		H	3.14
THR165[O]	TYR59[OH]	V_H		H	2.43
LYS166[NZ]	ASP32[O]	V_H		H	3.73
ILE130[O]	ARG112[NH1]	V_L	451.8	H	2.37
ARG131[NH1]	ARG108[O]	V_L		H	2.43
SER145[OG]	SER28[OG]	V_L		H	3.19
SER145[N]	SER30[OG]	V_L		H	3.67
ASN157[O]	ARG112[NH2]	V_L		H	3.44
ASN157[O]	ARG112[N]	V_L		H	3.55

[a]V_H: heavy chain variable region; V_L: light chain variable region.

[b]BSA: Buried surface area.

[c]H: Hydrogen bond; S: Salt bridge.

[d]Dist.: distance between the interacting atoms.

TABLE S7 Amino acids involved in the contacts between SD008 HA and C4H4 Fab (PISA).

Residues of SD008 HA[a]	Domain[b]	Residues of C4H4 Fab	Chain[c]
T126	RBD	D32, Y33	V_H
Y127	RBD	G108, E109	V_H
N128	RBD	D32, Y33, P34, H40, G107, G108, E109	V_H
G129	RBD	H40, E109	V_H
I130	RBD	E109	V_H
R131	RBD	L110	V_H
N157	RBD	W52, F56, N66	V_H
T157	RBD	W52, Q69	V_H
D157	RBD	Q69	V_H
N158	RBD	Q69, K72	V_H
A159	RBD	N66, Y67, Q69	V_H
A160	RBD	T63, N66	V_H
F161	RBD	N66	V_H
P162	RBD	F56, T58, N62, N66	V_H
Q163	RBD	T58, N60, N62	V_H
M164	RBD	Y59, N60	V_H
T165	RBD	Y59, N60	V_H
K166	RBD	D32, Y59	V_H
I130	RBD	R112	V_L
R131	RBD	I107, R108, E109, L110, R112	V_L
T132	RBD	Y36	V_L
N133	RBD	T31, Y36, R108	V_L
V135	RBD	T31, R108	V_L
S145	RBD	S28, S30 T31	V_L
S156	RBD	L110, R112	V_L
N157	RBD	T111, R112	V_L
T157	RBD	L110, T111	V_L
D157	RBD	L110, T111	V_L
K193	RBD	L110	V_L

[a]All the amino acids are shown according to H3N2 HA numbering.

[b]RBD: receptor binding domain.

[c] V_H: heavy chain variable region; V_L: light chain variable region.
